# Convergent Evolution and Host-Limiting Impacts of SARS-CoV-2 Revealed by Cellular Experiments

**DOI:** 10.1093/molbev/msaf274

**Published:** 2025-10-28

**Authors:** Ting Zhang, Ren-Rong Tian, Fengyi Li, Xiaolu Tang, Wenbin He, Zhen-Ping Hao, Lin Zhuo, Jian Lu, Xuemei Lu, Yong-Tang Zheng

**Affiliations:** State Key Laboratory of Genetic Evolution & Animal Models, Key Laboratory of Bioactive Peptides of Yunnan Province, KIZ-CUHK Joint Laboratory of Bioresources and Molecular Research in Common Diseases, Center for Biosafety Mega-Science, Kunming Institute of Zoology, Chinese Academy of Sciences, Kunming 650201, China; State Key Laboratory of Genetic Evolution & Animal Models, Key Laboratory of Bioactive Peptides of Yunnan Province, KIZ-CUHK Joint Laboratory of Bioresources and Molecular Research in Common Diseases, Center for Biosafety Mega-Science, Kunming Institute of Zoology, Chinese Academy of Sciences, Kunming 650201, China; State Key Laboratory of Genetic Evolution & Animal Models, Yunnan Key Laboratory of Biodiversity Information, Kunming Institute of Zoology, Chinese Academy of Sciences, Kunming, Yunnan 650223, China; Savaid Medical School and Future Technology School, University of Chinese Academy of Sciences, Beijing 100049, China; State Key Laboratory of Gene Function and Modulation Research, Center for Bioinformatics, School of Life Sciences, Peking University, Beijing 100871, China; State Key Laboratory of Genetic Evolution & Animal Models, Yunnan Key Laboratory of Biodiversity Information, Kunming Institute of Zoology, Chinese Academy of Sciences, Kunming, Yunnan 650223, China; State Key Laboratory of Genetic Evolution & Animal Models, Key Laboratory of Bioactive Peptides of Yunnan Province, KIZ-CUHK Joint Laboratory of Bioresources and Molecular Research in Common Diseases, Center for Biosafety Mega-Science, Kunming Institute of Zoology, Chinese Academy of Sciences, Kunming 650201, China; State Key Laboratory of Genetic Evolution & Animal Models, Key Laboratory of Bioactive Peptides of Yunnan Province, KIZ-CUHK Joint Laboratory of Bioresources and Molecular Research in Common Diseases, Center for Biosafety Mega-Science, Kunming Institute of Zoology, Chinese Academy of Sciences, Kunming 650201, China; Savaid Medical School and Future Technology School, University of Chinese Academy of Sciences, Beijing 100049, China; State Key Laboratory of Gene Function and Modulation Research, Center for Bioinformatics, School of Life Sciences, Peking University, Beijing 100871, China; Beijing Advanced Center of RNA Biology (BEACON), Peking University, Beijing 100871, China; State Key Laboratory of Genetic Evolution & Animal Models, Yunnan Key Laboratory of Biodiversity Information, Kunming Institute of Zoology, Chinese Academy of Sciences, Kunming, Yunnan 650223, China; Savaid Medical School and Future Technology School, University of Chinese Academy of Sciences, Beijing 100049, China; State Key Laboratory of Genetic Evolution & Animal Models, Key Laboratory of Bioactive Peptides of Yunnan Province, KIZ-CUHK Joint Laboratory of Bioresources and Molecular Research in Common Diseases, Center for Biosafety Mega-Science, Kunming Institute of Zoology, Chinese Academy of Sciences, Kunming 650201, China

**Keywords:** SARS-CoV-2, convergent evolution, spike protein, host-specific selective pressures, multihost experimental system

## Abstract

The ongoing pandemic caused by severe acute respiratory syndrome coronavirus 2 (SARS-CoV-2) has highlighted the virus's remarkable ability to evolve and adapt in diverse hosts. Despite the observation of recurrent mutations and convergent evolution in the viral genome, the mechanisms driving these processes remain poorly understood, particularly in the context of diverse host environments and limited genomic surveillance. We established a rigorously controlled in vitro cellular system within a Biosafety Level 3 Laboratory, ensuring strict adherence to biosafety protocols while passaging the virus in seven cell lines derived from four tissues across five mammalian species. High-throughput sequencing revealed consistent positive selection on the Spike (S) protein, highlighting its adaptability in the absence of adaptive immune responses or therapeutic pressures. Type I interferons (IFN-I) and APOBEC-mediated editing may emerge as key modulators of viral evolution. Notably, IFN-I activation is inversely correlated with the accumulation of S protein mutations (E484D, P812R/L, L1186R). Our findings uncover host-specific selective forces in shaping SARS-CoV-2 evolution and highlight the need for systematic approaches to mitigate viral transmission and emerging variants.

## Introduction

The ongoing pandemic caused by SARS-CoV-2 has had devastating global consequences (Zheng 2020). As a member of the *Betacoronavirus* genus, SARS-CoV-2 possesses a single-stranded positive-sense RNA (ssRNA+) genome (Yang and Rao 2021). While primarily associated with viral pneumonia, it has been demonstrated to infect various host tissues, leading to multiorgan dysfunctions (Puelles et al. 2020; Stein et al. 2022).

The zoonotic nature of SARS-CoV-2 has enabled its spread in a wide range of mammalian species, including *Homo sapiens*, *Mesocricetus auratus*, *Neovison vison*, and *Odocoileus virginianus* (Munir et al. 2020; Sia et al. 2020; Tarbuck et al. 2025). Comprehensive genomic surveillance is crucial for understanding the evolutionary dynamics of SARS-CoV-2 and the potential for cross-species transmission (Islam et al. 2021; Oude Munnink et al. 2021; Brito et al. 2022). Since its emergence, the virus has undergone numerous mutations, giving rise to variants of concern (VOCs) such as Alpha and Omicron, which have impacted disease severity and vaccine effectiveness (Yang et al. 2021; Andre et al. 2023; Markov et al. 2023; Yao et al. 2024). The emergence of certain VOCs has been arguably linked to the undetected spread of the virus in regions with limited genomic surveillance in both human and animal populations (Markov et al. 2023). The detection of cross-species transmitted mutations, such as N501Y and E484D in the receptor binding domain (RBD) of S protein, underscores the potential threat of viral reverse-spillover from animal reservoirs to humans (Zhou et al. 2021; Baggen et al. 2023).

Convergent evolution, characterized by the independent emergence of similar traits or genetic changes in different lineages under comparable selective pressures, has been observed in SARS-CoV-2, particularly in the form of recurrent mutations in the N-terminal domain (NTD) and RBD of the S protein, which are critical for viral entry and immune evasion (Cao et al. 2022; Yao et al. 2024). Specific mutations, such as N501Y and K417N in the RBD, and D128Y in the nucleocapsid protein (N), have been identified as shared between humans and animals, linked to enhanced immune evasion or increased transmission and replication (Zhou et al. 2021; Yao et al. 2024). However, the underlying mechanisms and driving forces behind the convergent evolution in SARS-CoV-2 remain poorly understood, likely due to the complex interplay among viral genetics, host immune responses, and environmental factors, as well as the lack of comprehensive genomic surveillance in certain regions and host species.

The evolutionary dynamics of SARS-CoV-2 have been examined from multiple perspectives, including RNA replication errors, recombination, APOBEC-mediated C>U editing, and natural selection (Qian et al. 2022; Markov et al. 2023; Roemer et al. 2023). Additionally, factors such as receptor binding, tissue pressure, antigenic drift, and immune escape facilitate the circulation of advantageous mutations (Markov et al. 2023). The innate interferon response, particularly type I interferons (IFN-I), serves as the first line of defense against viral infections by triggering the transcription of IFN-stimulated genes (ISGs) (Schneider et al. 2014; Schoggins 2019; Streicher and Jouvenet 2019). This antiviral signaling cascade operates in various cell types exposed to IFN-I (Crouse et al. 2015; Makris et al. 2017). In response, SARS-CoV-2 has developed strategies to evade the host immune surveillance, including antagonizing IFN production, inhibiting IFN signaling, and enhancing IFN resistance (Xia et al. 2020; Minkoff and tenOever 2023). However, existing studies on the impact of IFN-I-mediated selective pressure on SARS-CoV-2's evolution have been limited in scope and scale, underscoring the need for a more systematic investigation.

In essence, a significant knowledge gap exists in understanding how SARS-CoV-2 adapts in different host species at the cellular level, particularly concerning its evolutionary dynamics and the influence of host-specific factors on mutation patterns. Previous research has focused primarily on SARS-CoV-2 in human populations or single-host models, leaving unresolved questions about the convergent evolution and the shared evolutionary pressures among diverse species. Additionally, while the roles of IFN-I responses and APOBEC enzymes in viral replication are recognized, their specific contributions to shaping SARS-CoV-2's mutation patterns and adaptation in species remain inadequately explored, especially in multispecies contexts. Furthermore, a lack of experimental evidence limits our understanding of the virus's cross-species adaptability and the host-specific constraints that influence its evolution.

Here, we established well-controlled in vitro cellular systems under strict biosafety management within a certified Biosafety Level 3 Laboratory (BSL-3), enabling systematic monitoring of SARS-CoV-2 evolution in four different tissues (lung, liver, kidney, and colon) from five species (*Homo sapiens*, *Macaca mulatta*, *Chlorocebus sabaeus*, *Mesocricetus auratus*, and *Felis catus*) using high-throughput sequencing. This carefully regulated approach allows us to track the evolving mutation landscape of SARS-CoV-2 in diverse tissues and species contexts, revealing convergent mutations shaped by distinct evolutionary pressures and constraints. Furthermore, we identified APOBECs and IFN-I pathways as significant factors that shape the mutations and adaptation of SARS-CoV-2 in different cellular systems.

## Results

### Establishing Serial Passage Models for SARS-CoV-2 in Diverse Cell Lines

**Fig. 1.**
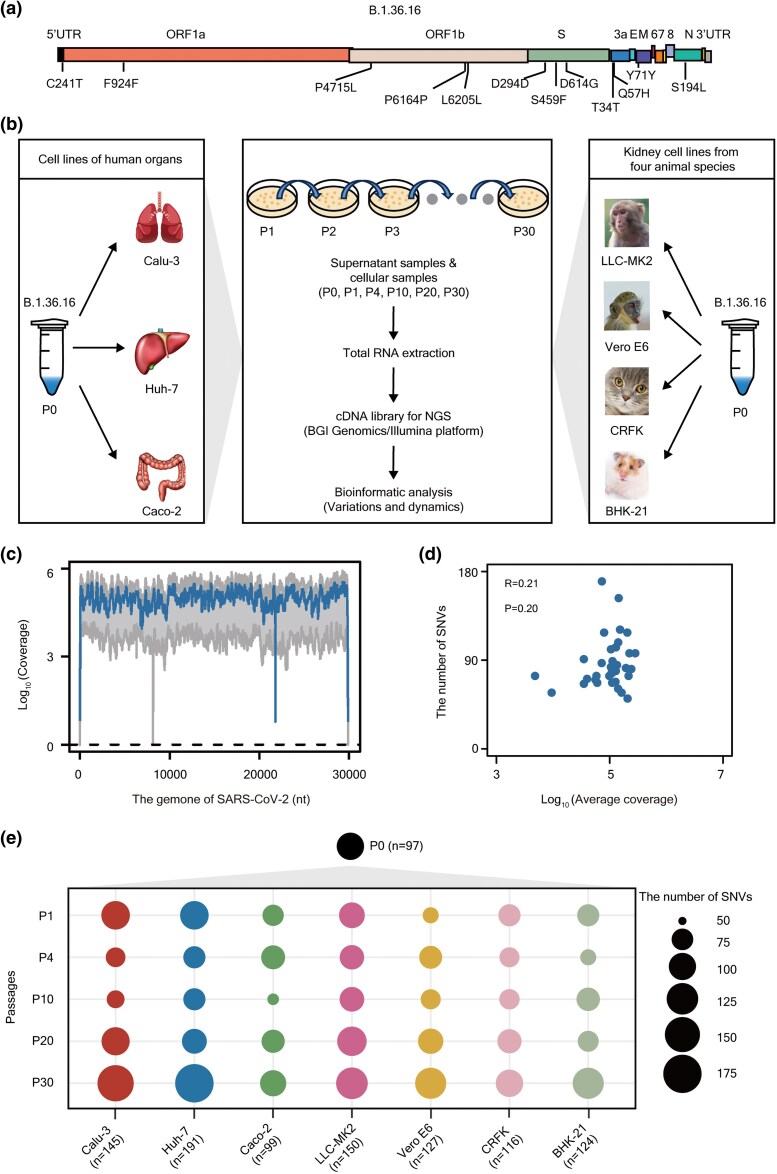
The serial passage scheme of SARS-CoV-2 in cell lines derived from different organs and species. a) The SARS-CoV-2 strain used in this study contains 12 SNPs compared to the reference genome. b) Passaging and sequencing strategy of SARS-CoV-2 in seven cell lines used in this study. Three cell lines were derived from various human organs, including the lung (Calu-3), liver (Huh-7), and colon (Caco-2). The other four cell lines were kidney-derived from different animal species, including *Macaca mulatta* (LLC-MK2), *Chlorocebus sabaeus* (Vero E6), *Felis catus* (CRFK), and *Mesocricetus auratus* (BHK-21). For each cell line, the virus was passaged sequentially, and the supernatant and cellular RNAs of certain passages were, respectively, collected. Total RNA was extracted from the samples of certain passages (P0, P1, P4, P10, P20, P30) and used to prepare cDNA libraries for next-generation sequencing using the BGI Genomics or Illumina platform. Moreover, bioinformatic analyses were conducted to detect variations and changes in the viral genome. c) Line plots showing the genome coverage of the sequenced samples across the SARS-CoV-2 genome. The blue line represents the median sequencing depth, and the grey region represents the minimum and maximum of the sequencing depth. d) Dot plot showing the *Pearson* correlation (*R*) between the average sequencing depth and the number of identified SNVs for each sample. The statistical significance was calculated by *Pearson*'s test. e) Bubble plot illustrating the number of SARS-CoV-2's SNVs identified during the indicated passage in the indicated cell line. The size of each bubble represents the number of SNVs identified, with larger bubbles indicating more SNVs. *N* represents the total number of SNVs identified across all passages for each cell line after deduplication. The threshold used for SNV identification is sequencing depth ≥ 100 and MuAF ≥ 0.01.

We developed serial passage models to study SARS-CoV-2 by infecting various cell lines from different species. The SARS-CoV-2 isolate used in these experiments, referred to “strain T” in this study, was isolated from a patient in September 2020, and belongs to the B.1.36.16 lineage, a derivative of the dominant B.1.36 strain from the first wave of the pandemic (Basheer and Zahoor 2021). Compared to the reference genome (GenBank: NC_045512.2), strain T harbors 12 single-nucleotide polymorphisms (SNPs) (Fig. 1a), including notable mutations such as C241T, C3037T, C14408T, and A23403G|S:D614G, which emerged in mid-February 2020 and rapidly became dominant (Yang et al. 2021).

We passaged strain T in seven cell lines derived from five different species (Fig. 1b and Table S1) under stringent biosafety control in a certified BSL-3. These cell lines were selected to represent various human tissues, including lung (Calu-3), liver (Huh-7), and colon (Caco-2), as well as kidney cell lines from four different animal species: *Macaca mulatta* (LLC-MK2), *Chlorocebus sabaeus* (Vero E6), *Felis catus* (CRFK), and *Mesocricetus auratus* (BHK-21). Five of these cell lines have been reported to be highly susceptible to SARS-CoV-2 infection (Calu-3, Huh-7, Caco-2, LLC-MK2, and Vero E6) (Hoffmann et al. 2020; Wurtz et al. 2021; Wang et al. 2021a), while CRFK and BHK-21 cells showed lower vulnerability to this virus (Hoffmann et al. 2020; Wurtz et al. 2021; Wang et al. 2021a). By passaging the virus through multiple cell lines in parallel, we compared evolutionary trajectories, revealed adaptive mutations, and identified host-specific factors that influence SARS-CoV-2's evolution. Using diverse cell lines from multiple species and tissues provides convenient, rapid, and high-throughput in vitro models for studying SARS-CoV-2's adaptability and evolutionary dynamics.

To explore the genetic basis of SARS-CoV-2's fitness changes, we passaged strain T in these cell lines, analyzing the cytopathic effects (CPEs) during each propagation and characterizing mutational landscapes of SARS-CoV-2 by deep sequencing the supernatant RNA from a shared P0 stock (the starting inoculum) and five passage generations (P1, P4, P10, P20, and P30) for each infected cell line (Fig. 1b). The multiplicity of infection (MOI) used for each cell type was optimized to balance viral load, CPE progression, and long-term propagation efficiency. We successfully obtained deep sequencing results for 36 samples, yielding 752 single-nucleotide variants (SNVs), including 97 detected in P0 at a sequencing depth ≥ 100 and mutant allele frequency (MuAF) ≥ 0.01 (Fig. 1c and d). Applying a more stringent MuAF threshold (≥ 0.05) identified 191 SNVs across all samples. Although the number of identified SNVs varied across cell types, a consistent increase was observed in nearly all cell lines over time at both thresholds (Fig. 1e and Figure S1a).

### SARS-CoV-2's Adaptation Increases Its CPE and Replication Across Cell Lines

**Fig. 2.**
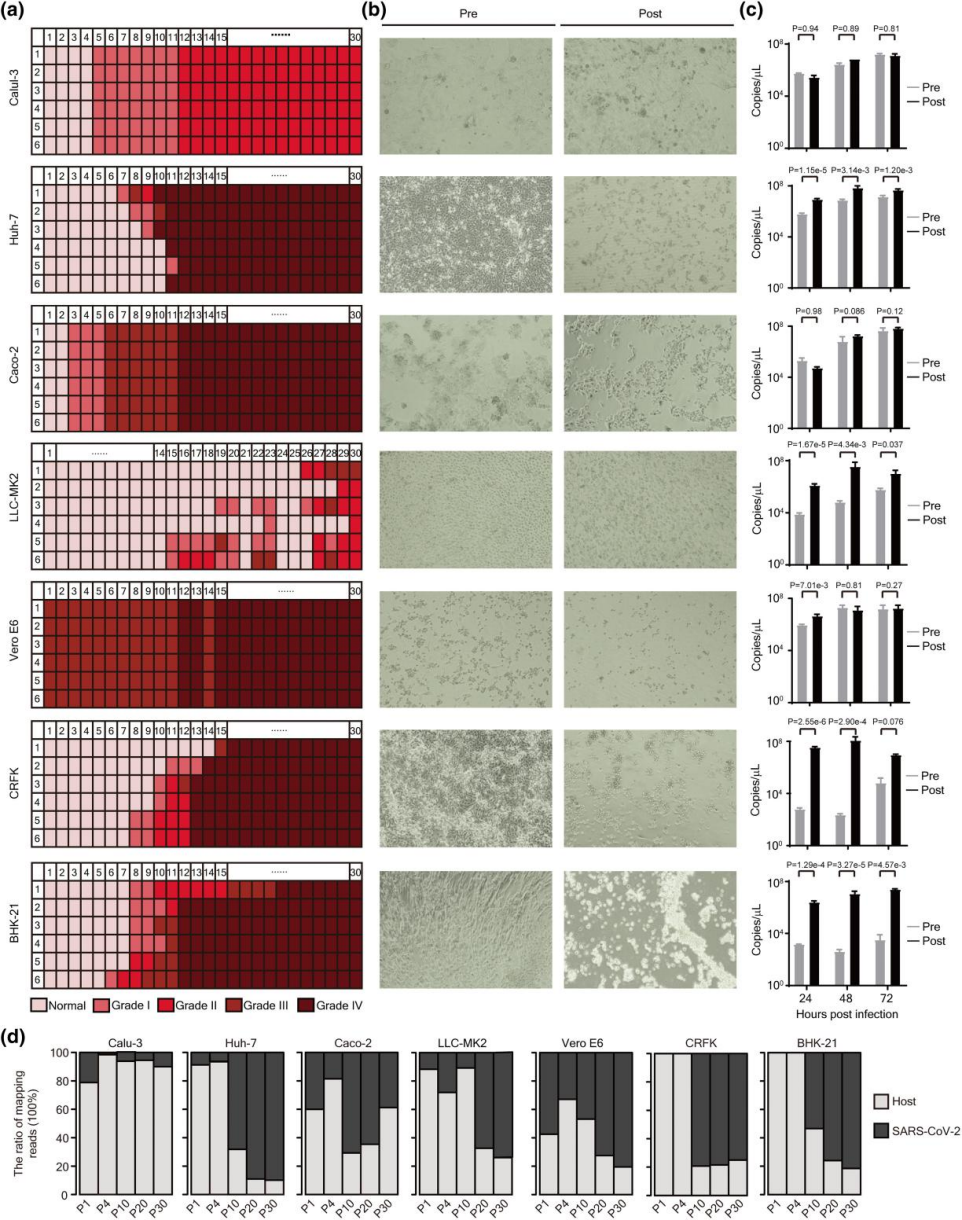
Adaptation of SARS-CoV-2 to efﬁcient growth in different organ-derived cell lines of different species. a) Heatmaps showing the cellular pathological level of the indicated cells. Calu-3, Caco-2, Huh-7, Vero E6, LLC-MK2, CRFK, and BHK-21 cells were initially infected with SARS-CoV-2 at the MOI of 0.05, 0.05, 0.2, 0.01, 0.4, 0.4, and 0.4 for 2 h, followed by the medium was replaced. After 3 days' culture, the supernatants from Calu-3, Caco-2, Huh-7, Vero E6, LLC-MK2, CRFK, and BHK-21 were individually diluted 2, 10, 10, 200, 1, 1, 1 time, respectively, to infect the corresponding normal cells. Following an incubation period of 2 h, the medium was changed, and the culture was continued for 3 additional days. Then, the supernatant was collected for the next round. Before harvesting the supernatant at each passage, cellular pathology, such as rounding, detachment, wrinkling, rupture, and cell death, was conducted. Cellular pathology was categorized into four grades based on the quantitative assessment of cell changes. The passages and observations were performed until the 30th generation (*n* = 6). b, c) The adaptive changes of SARS-CoV-2 in (b) cell morphological and (c) viral replication efficiency. Equal MOIs of P0 and P30 virus were used to infect Calu-3, Caco-2, Huh-7, Vero E6, LLC-MK2, CRFK, and BHK-21 cells, at the MOIs of 0.5, 0.5, 0.5, 0.01, 0.5, 0.5 and 0.5, respectively. Following 2 h’ incubation with virus, the cells were washed with phosphate buffered saline (PBS) for three times and then incubated for 72 h. Cell morphology was examined, and photographs were captured at 72 h after infection. The supernatants at 24, 48, and 72 h after infection were collected, and the nucleic acids from them were extracted, followed by viral detection by qRT-PCR (*n* = 3). The statistical significance was calculated by one-tailed student's *t*-test. d) Bar plots displaying the proportion of unique mapping reads allocated to the host (in light grey) versus the SARS-CoV-2 genome (in dark grey) at the specified passage in the indicated cell.

As shown in Fig. 2a, SARS-CoV-2 exhibited no CPEs during the first four passages in most cell lines, with moderate effects observed in Caco-2 and Vero E6 cells. However, during passages 5 to 12, CPEs increased in nearly all cell types. By the 30th passage, approximately 75% of cells exhibited signs of damage, such as rounding, detachment, or cell death. However, we observed relatively mild effects in all the six replicates of Calu-3 cells.

To assess the impact of adaptation on viral replication and CPEs, we infected each cell line with both the starting inoculum (P0, preadaptation) and the corresponding cell line-adapted strains (P30, postadaptation) of SARS-CoV-2 at equivalent titers and monitored changes in cell morphology and viral loads in the supernatant. After adapting to various human organ-derived cell lines, SARS-CoV-2 showed a significant increase in CPEs, especially in Huh-7 and Caco-2 cells, with replication efficiency in Huh-7 cells increasing by 13 times (Fig. 2b and c). Similar increases in CPEs were observed in diverse animal kidney cell lines, with replication efficiency increasing by 4.9, 56624.8, 166.8, and 1,786.2-fold in Vero E6, CRFK, LLC-MK2, and BHK-21 cells, respectively (Fig. 2b and c).

To assess viral levels during culture and passaging, we compared the abundance of SARS-CoV-2 mRNAs relative to host mRNAs in the mRNA-Seq data. Fig. 2d illustrates that the differences in CPEs among various cell types during passages are closely linked to the viral abundance detected in these cells. For instance, it is generally acknowledged that Vero E6 exhibits strong CPEs due to: 1) higher expression levels of ACE2 and the use of CTSL instead of TMPRSS2, which facilitate faster viral entry (Koch et al. 2021); and 2) a deficiency in IFN-I (Geerling et al. 2022), resulting in weak antiviral activity and allowing easy viral replication. Indeed, we found that the virus replicates more rapidly in Vero E6 than in other cell lines from P1 to P10. Moreover, we observed that Calu-3 cells experience moderate infection during the passaging process, which corresponds well with the relatively mild effects noted in all six replicates of Calu-3 cells.

In summary, these results demonstrate that SARS-CoV-2 can rapidly adapt to different cell types, leading to enhanced CPEs or increased replication efficiency. Notably, the marked increases in both parameters in Huh-7 cells suggest that the virus can easily adapt to human liver cells, potentially increasing its capacity to cause systemic infections. Likewise, the substantial increases in CPEs and replication observed in diverse animal kidney cell lines, including Vero E6 and LLC-MK2, and even in CRFK and BHK-21 cells that showed lower susceptibility to the viral infection (Hoffmann et al. 2020; Wurtz et al. 2021; Wang et al. 2021a), underscoring the virus's capacity to cross species barriers and adapt to new hosts rapidly.

### Positive Selection on the *S* Gene During SARS-CoV-2's Adaptation

**Fig. 3.**
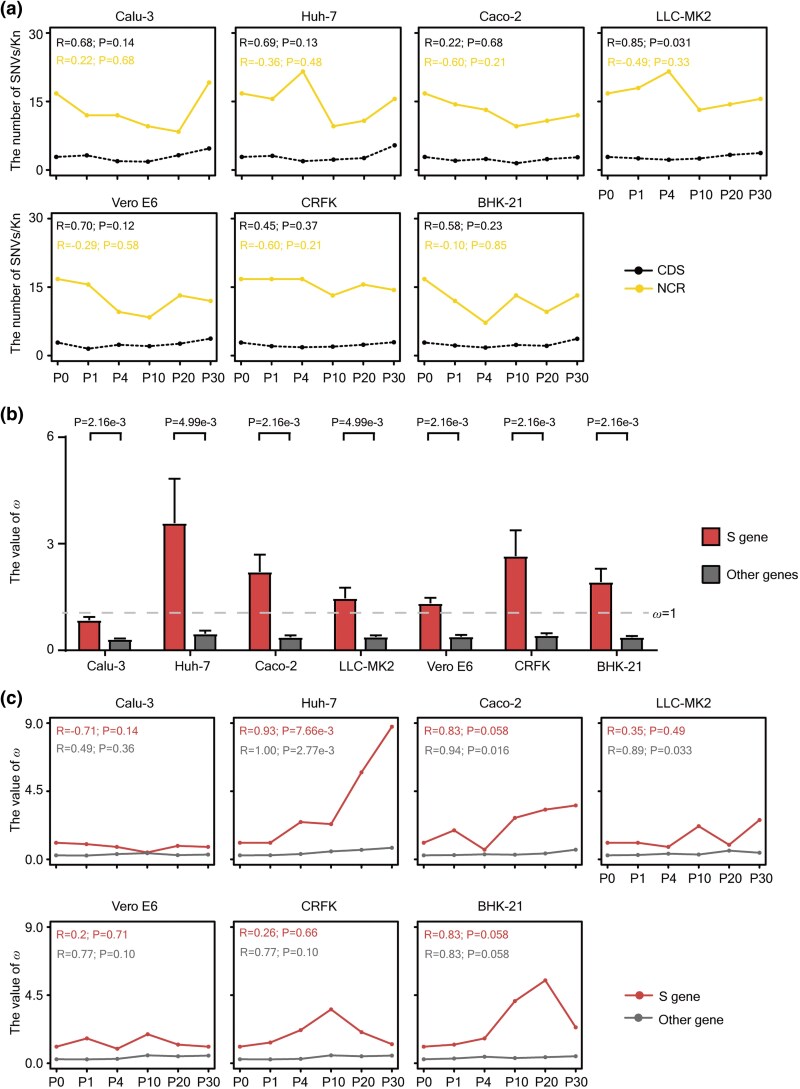
Positive selection drives the adaptive evolution of the S gene in SARS-CoV-2. a) Line plots displaying the temporal change of the number of SNVs per kilobase in CDS (in black) and NCR (in gold) in the indicated cell line. The relationship between the normalized count of SNVs and the passages was evaluated using *Pearson*'s test, with *R* denoting the *Pearson* correlation coefficient. b) Bar plots showing the *ω* values for different genes in the indicated cell line. As shown, the *ω* value was significantly higher in the *S* gene (in red) than in other genes (in grey) during passages in each cell line (*n* = 6). The dash grey line represents the *ω* = 1, and the statistical significance was calculated by unpaired two-tailed Wilcoxon signed rank test. (c) Line plots displaying the temporal trend of the *ω* values in different genes in the indicated cell line. The correlation between the *ω* values and passages was analyzed using *Spearman*'s test with *R* representing the *Spearman* correlation coefficient.

Analysis of the SNV (MuAF ≥ 0.01) distribution in the viral genome revealed that 75.70% to 93.10% of the SNVs were located within coding sequences (CDS), which comprise about 97.00% of the genome (Figure S2a). After normalizing for sequence length, the density of SNVs per 1,000 nucleotides (SNVs/Kn) was higher in noncoding regions (NCRs) than in CDS (Fig. 3a). Although this difference was not statistically significant, the trend is consistent with the hypothesis that NCRs may be under weaker purifying selection than CDS. Notably, a temporal increase in nonsynonymous SNVs (NS) was observed in nearly all cell lines, while synonymous SNVs (Syn) showed dynamic patterns, likely reflecting positive selection for NS mutations across tissues and species (Figure S2a).

Mutations in the SARS-CoV-2 *S* gene, particularly within the RBD, have commonly been linked to enhanced ACE2 receptor binding, immune evasion, or increased infectivity in ACE2-negative cells (Baggen et al. 2023; Chen et al. 2024; Liu et al. 2024; Yao et al. 2024). These mutations complicate the development of vaccines and monoclonal antibodies (Noh et al. 2021; Wang et al. 2021b; Luo et al. 2022). The Ka/Ks ratio (*ω*), defined as the rate of nonsynonymous (Ka) to synonymous (Ks) substitutions, is commonly used to infer selective pressure during long-term interspecies divergence (Nei and Gojobori 1986). Although this measure can be biased by bottlenecks, drift, and transient polymorphisms in short-term cell-culture evolution, we used Ka/Ks as a relative indicator across genes and cell lines rather than as an absolute test of selection. For each passage in each cell type, we counted NS and Syn with MuAF ≥ 0.01 and calculated Ka, Ks, and *ω* for the *S* gene and for the concatenated CDSs of the remaining genes. Consistently, *ω* for the *S* gene was higher than for most other genes across cell lines (Fig. 3b), in line with previous reports of positive selection acting on *S* (Tang et al. 2023). Notably, the *ω* value of the *S* gene increased over time in Huh-7, Caco-2, LLC-MK2, Vero-E6, CRFK, and BHK-21 cells (Fig. 3c), while a dynamic decrease was observed in Calu-3 cells during passaging, which might be related to the activation of the interferon pathway (see below).

**Fig. 4.**
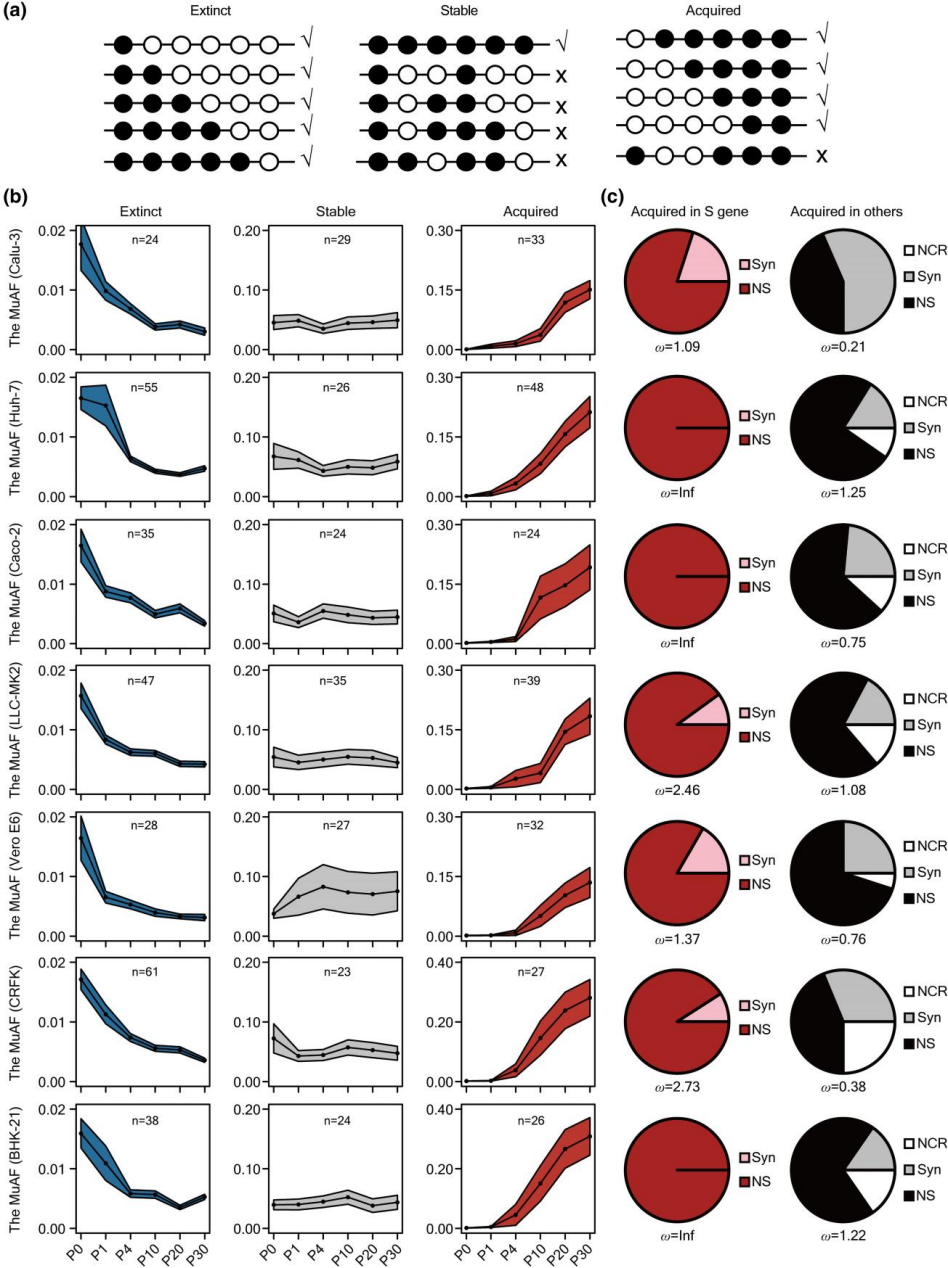
The temporal characteristics of SARS-CoV-2’s SNVs in different clusters. a) Workflow of clustering SNVs into extinct (left panel), stable (middle panel), or acquired groups (right panel). b) Clustering for the SNVs of SARS-CoV-2 detected in the indicated cell line across passages. According to the change of MuAF during passages, SNVs have been divided into three groups, namely extinct (left panel), stable (middle panel), and acquired (right panel). Data are represented by MuAF with mean ± SEM. *N* represents the number of SNVs in the indicated cluster. (c) Pie charts illustrating the ratio of the acquired SARS-CoV-2's SNVs identified as NCR, Syn, and NS within the *S* gene (left panel) or other regions (right panel). The *ω* value for the *S* gene and the other genes has also been calculated.

Based on MuAF profiles across passages, SARS-CoV-2 SNVs were classified into three categories: extinct (present in P0 but lost in later passages), stable (persisting through all passages), or acquired (emerging after P1 and persisting). For each cell type, only mutations with MuAF ≥ 0.01 and sequencing depth ≥ 100 in at least one passage were considered (Fig. 4a). The analysis revealed that 21.62% to 70.11% of SNVs detected at P0 were lost during propagation in different cell lines, while 27.90% to 31.53% were stable. The proportion of acquired SNVs, which appeared at P1 and persisted in subsequent passages, ranged from 18.60% to 43.24% (Fig. 4b). The average MuAF remained consistently low (<0.02) for the extinct type, while it consistently approximated 0.1 for the stable type in all examined cell lines. However, the acquired class generally showed increasing MuAF during passaging. Linear regression analyses of the acquired mutations in a cell type revealed that every mutation (100%) displayed a positive correlation (*R* > 0) with passage number, consistent with gradual frequency increases over time (Table S2). Furthermore, significant positive trends (*P* < 0.05) were detected for the majority of acquired mutations in each cell line: Calu-3 (72.73%), Huh-7 (66.67%), Caco-2 (66.67%), LLC-MK2 (53.85%), Vero E6 (78.12%), CRFK (85.19%), and BHK-21 (69.23%). For the acquired class in each cell type, we performed Cochran–Mantel–Haenszel (CMH) to compare the ratio of NS to Syn (NS/Syn) in the *S* gene versus all other concatenated genes. The CMH test results revealed a statistically significant association (*P* = 5.07 × 10^−4^) between gene category (*S* gene vs. other genes) and mutation type (NS vs. Syn), indicating that NS are enriched in the *S* gene within the acquired mutation class (Fig. 4c).

A caveat in the previous analysis is that we primarily focused on variants with MuAF ≥ 0.01. Variants with lower MuAF are less likely to be under selection and may be disproportionately affected by random noise, including sequencing and mapping errors, as well as sampling bias. To further substantiate our conclusions, we also assessed SNVs using a more stringent MuAF threshold (≥ 0.05). Similarly, we detected a significant temporal increase in C>U mutations in all cell lines (Figure S1b and c). Despite the limited number of mutation sites precluding value calculation at certain passages, analysis of the P30 samples continued to indicate significant positive selection acting on the S protein in all cell lines except for Calu-3 (Figure S1d). Collectively, these results reinforce our conclusion that positive selection on the *S* gene intensifies over time in most cell types, except for Calu-3, and that this pattern holds under both MuAF ≥ 0.01 and MuAF ≥ 0.05 thresholds. In subsequent analyses, we will focus on SNVs with MuAF ≥ 0.01, while applying stricter cutoffs when necessary to validate key evolutionary patterns.

### APOBEC-mediated RNA Editing Potentially Induces C > U Mutational Bias in SARS-CoV-2 Across Cell Lines

**Fig. 5.**
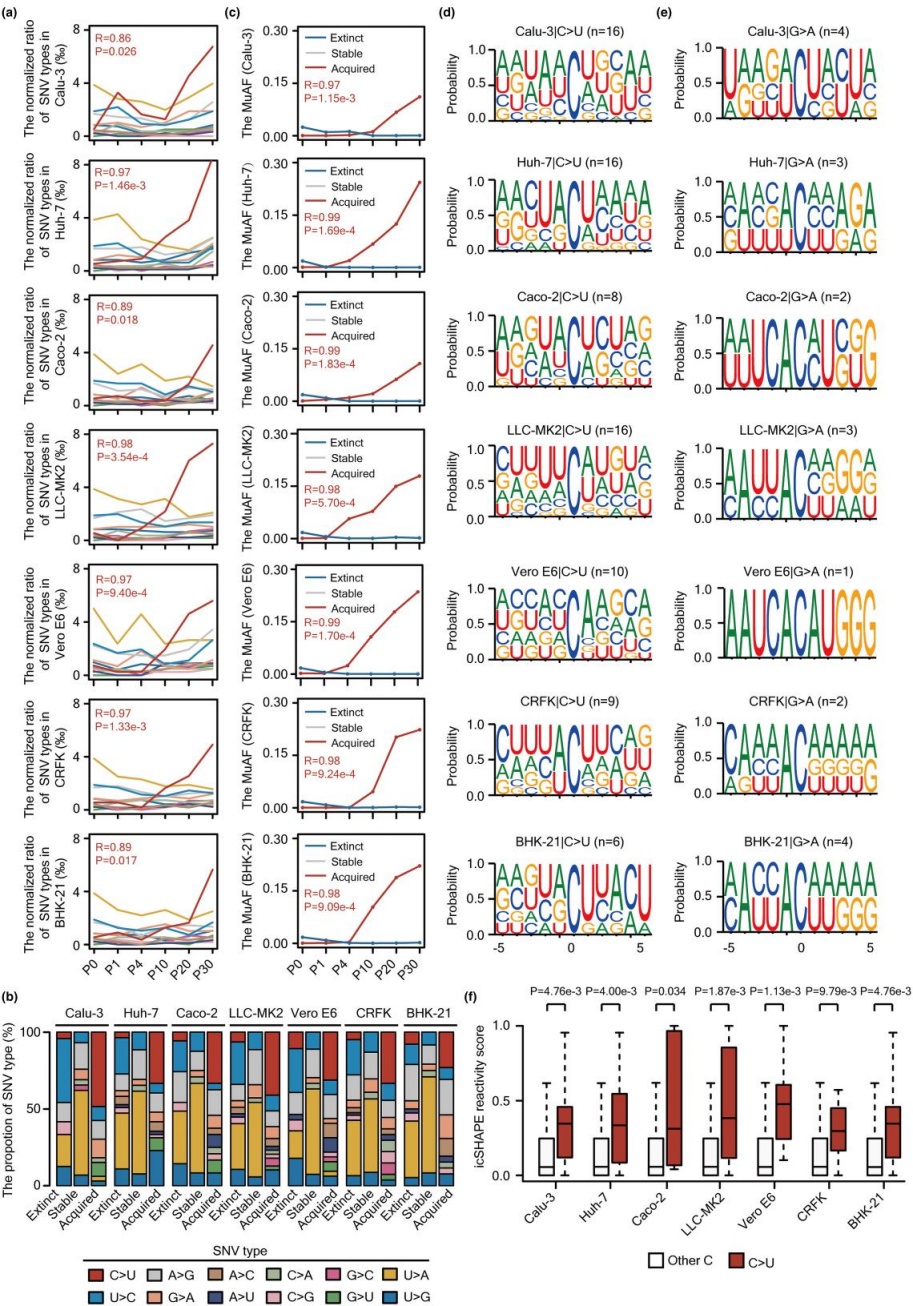
APOBEC activity is a potential source of C>U mutation bias in SARS-CoV-2 during passage in different cell lines. a) Line plots illustrating the normalized ratio of 12 types of mutations of SARS-CoV-2 at each passage in the indicated cell line. At each passage, the number of each type of mutation was normalized by the number of the corresponding nucleotide contained in the SARS-CoV-2 genome (i.e. the number of C>U SNVs/the number of Cs in the viral genome). The *Pearson* correlation coefficient (*R*) for the normalized ratio of C>U mutations (in red) and passages is labeled, whereas other types are shown in Figure S2d. The statistical significance was calculated by *Pearson*'s test. b) Bar plots showing the frequency of twelve mutation types of SARS-CoV-2 in the extinct, stable, or acquired groups. The SNVs were, respectively, detected in the indicated cell line. c) Line plots illustrating the average MuAF for C>U substitutions of SARS-CoV-2 in the extinct (in blue), stable (in grey), and acquired (in red) categories of each cell line. The *Pearson* correlation coefficients (*R*) for the average MuAF of the acquired C>U SNVs in relation to passages have been computed and labeled. The statistical significance was calculated by *Pearson*'s test. d, e) Sequence motifs of the acquired (d) C>U or (e) G>A SNVs of SARS-CoV-2 with flanking ± 5 nucleotides in the indicated cell line. For G>A, the reverse complementary sequences have been used. f) Distribution of the in vivo icSHAPE reactivity scores at the C sites, with (in red) or without (in white) acquired C>U mutations in the indicated cell line. The icSHAPE reactivity scores were measured by Sun et al. (2021). The statistical significance was calculated by unpaired one-tailed Kolmogorov–Smirnov test.

In the initial (P0) SARS-CoV-2 population, we observed 41 transitions (Ti) and 56 transversions (Tv) relative to the reference genome, yielding a Ti/Tv ratio of 0.73. This ratio increased with serial passaging, surpassing 1.2 in all tested cell lines and peaking at 1.36 in Huh-7 (*R* = 0.90, *P* = 0.012) and 1.69 in LLC-MK2 (*R* = 0.89, *P* = 0.017) (Figure S2b). Among Ti mutations, C>U substitutions rose markedly in frequency (from 3.09% to 26.69%, Figure S2c), a trend that remained significant after normalization for base composition (Fig. 5a and Figure S2d). C>U mutations accounted for < 10.00% of extinct SNVs and were absent among stable variants. In contrast, it comprised > 23.00% of acquired SNVs (Fig. 5b), and its average MuAF increased significantly over time (Fig. 5c), underscoring a pervasive C>U bias during viral adaptation.

Given the known role of APOBECs in catalyzing C>U deamination on ssRNA (Di Giorgio et al. 2020; Ratcliff and Simmonds 2021; Liu et al. 2022; Nakata et al. 2023), we examined local sequence context. Both C>U and G>A mutations were enriched for U/A/C at the −1/+1 positions (Fig. 5d and e), consistent with APOBEC motifs (Di Giorgio et al. 2020; Pecori et al. 2022). The C sites undergoing C>U mutations in each cell line were predominantly found in ssRNAs, as indicated by their elevated icSHAPE reactivity scores (Sun et al. 2021) relative to other cytosines (Fig. 5f). Together, these data implicate APOBEC activity, preferentially targeting the positive-sense strand, potentially causing C>U mutational bias during SARS-CoV-2 adaptation across diverse cell lines. The significant enrichment of these mutations in the acquired variants (Fig. 5b and c), coupled with their predominance in circulating strains in human populations (Di Giorgio et al. 2020; Rice et al. 2021), suggests that they may shape viral evolutionary trajectories, although their adaptive significance requires functional validation.

In contrast, A>G and U>C mutations, potentially mediated by ADARs on dsRNA (Pecori et al. 2022), occurred at similar levels across cell lines (Figure S3a), but lacked enrichment for canonical ADAR motifs (Pecori et al. 2022) or structured RNA regions (Figure S3b–d). These findings argue against a prominent role for ADARs in shaping SARS-CoV-2 mutational spectra under these conditions.

### Abundant Convergent Mutations During SARS-CoV-2's Adaptation to Diverse Cell Lines

**Fig. 6.**
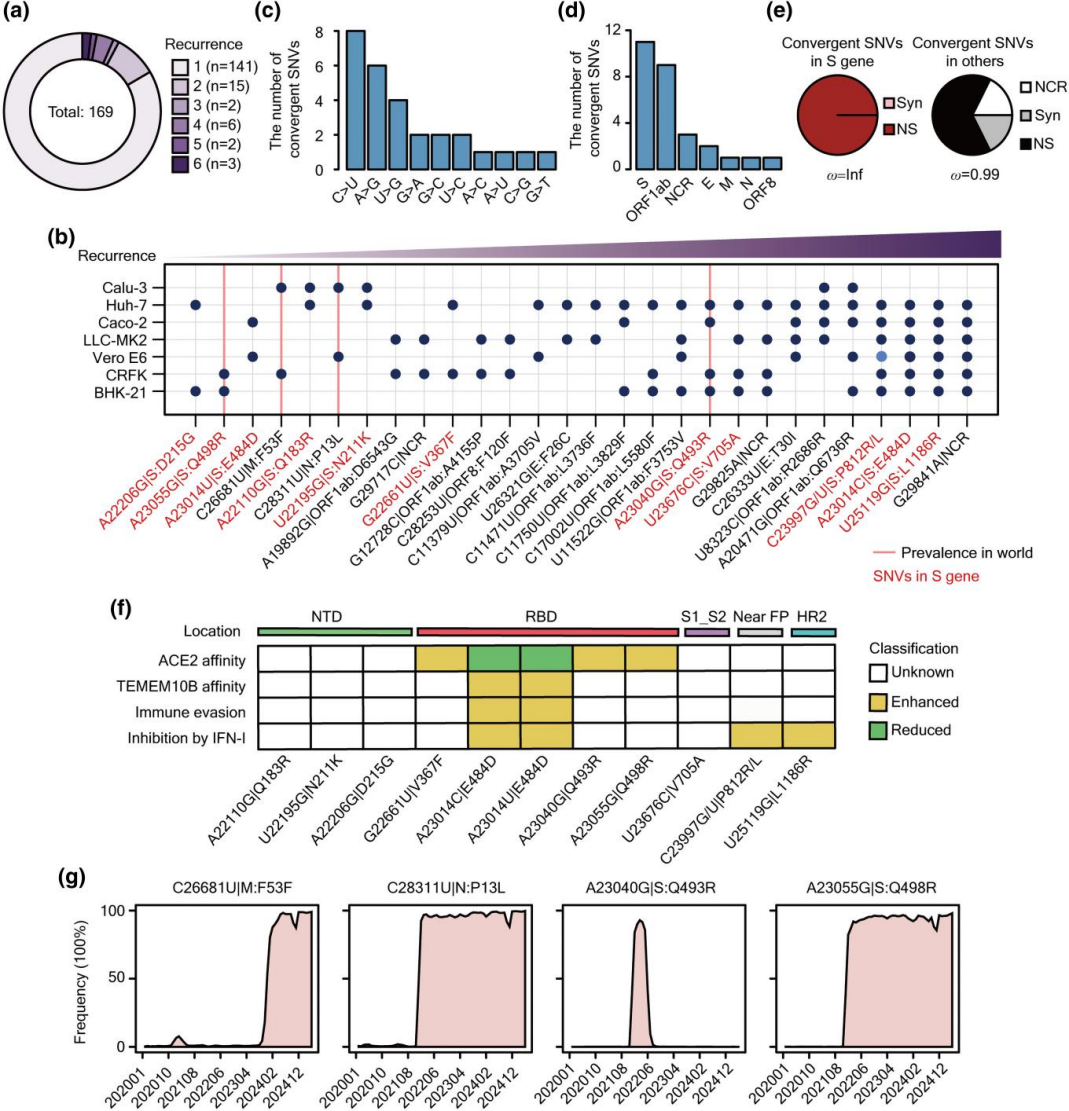
The characteristics of convergent SNVs. a) Pie plot showing the ratio of the acquired SNVs of SARS-CoV-2 determined by their recurrence across cell lines. After analysis, 28 acquired SNVs have been discovered in ≥ 2 distinct cell lines and designated as convergent sites. b) Dot plot showing the recurrence detail of 28 convergent SNVs. Blue dots represent the convergent SNVs acquired in the indicated cell line after 30 passages. The convergent SNVs distributed in *S* gene of SARS-CoV-2 have been marked in red. Red lines represent the SNVs with global prevalence in populations. At the 23997th position of C, two types of mutations were found, one of which is C23997G|S:P812R. This mutation appeared in all cells except Calu-3 and Vero E6. In addition, C23997U|S:P812L was found only in Vero E6 cells and is indicated with the light blue dot. c, d) Bar plots depicting the numbers of convergent sites distributed in (c) different types of mutations or (d) different viral genomic regions. e) Pie charts displaying the ratio of the convergent SARS-CoV-2's SNVs identified as NCR, Syn, and NS within the *S* gene (left panel) or other regions (right panel). The *ω* values of different genes have been calculated and labeled. f) Heatmap displaying the established biological roles associated with the 11 convergent sites distributed in S protein. The white square represents an unknown function, the green square represents a weakened function, and the yellow square represents an enhanced function. g) Line plots showing the SNVs' prevalence over time at C26681U|M:F53F, C28311U|N:P13L, A23040G|S:Q493R, and A23055G|S:Q498R. The sequence data were obtained from the GISAID database (as of Jun 5, 2025).

Convergent evolution in viruses pinpoints critical mutations that independently arise in different strains, enhancing viral fitness and adaptability to diverse hosts and environments (Zhou et al. 2021; Upadhyay et al. 2022 ; Focosi et al. 2023 ). Among the mutations acquired during viral propagation in cell lines, 169 SNVs were also detected in SARS-CoV-2 variants circulating in human populations (Table S3). It demonstrates that our system is capable of effectively detecting and identifying mutations pertinent to the ongoing evolution of SARS-CoV-2 (Figure S4). Among them, 28 mutations have recurred in two or more cell lines, which are defined as convergent SNVs (Fig. 6a and b). Among these, C>U mutations were the most frequent (8 SNVs), followed by A>G mutations (6 SNVs), and these convergent SNVs are unevenly distributed in the *ORF1ab* (9 SNVs), *S* (11 SNVs), *E* (2 SNVs), *M* (1 SNVs), *N* (1 SNVs), *ORF8* (1 SNVs), and NCR (3 SNVs) regions of SARS-CoV-2 (Fig. 6c and d). Notably, the *ω* value of the convergent SNVs in the *S* gene is higher than other genes, suggesting a stronger signature of positive selection (Fig. 6e). In particular, two convergent SNVs (A23014C|S:E484D and U25119G|S:L1186R) were observed in six cell lines, with the exception of Calu-3 cells. Interestingly, we identified two variants at nucleotide C23997, namely C23997G|S:P812R and C23997U|S:P812L. Variant C23997U was exclusively observed in Vero E6 cells (Fig. 6b and Figure S5), while C23997G was found in Huh-7, Caco-2, LLC-MK-2, CRFK, and BHK-21 cells.

Among the 11 convergent SNVs in the *S* gene in multiple cell lines, 5 are located in the RBD (G22661U|V367F, A23014U|E484D, A23014C|E484D, A23040G|Q493R, and A23055G|Q498R), 3 in the NTD, and others near the S1/S2 junction, fusion peptide, and heptad repeat 2 (HR2) (Fig. 6f). All these RBD mutations have been validated to enhance SARS-CoV-2's infectivity or transmission by affecting ACE2 binding affinity (Ou et al. 2021; Hong et al. 2022; Baggen et al. 2023; Philip et al. 2023).

Two further convergent SNVs at nucleotide A23014 (A>C and A>U) change glutamate (E) to aspartate (D) at residue 484 of the S protein (Fig. 6b and Figure S5). This amino acid change, occurring in six cell lines except Calu-3, can promote SARS-CoV-2's infection in ACE2-negative cells via the TMEM106B pathway (Baggen et al. 2023). It can also enhance viral tissue tropism, interspecies transmission, and resistance to antibody neutralization (Hoffmann et al. 2022; Baggen et al. 2023). In conclusion, these convergent mutations, particularly in the *S* gene, enhance SARS-CoV-2's adaptability and evolution in diverse hosts, highlighting their importance in viral evolution and the ongoing pandemic response. The findings underscore the critical role of the RBD mutations in adapting SARS-CoV-2 in different species.

### Overlapped SNVs From Experimental Cell Evolution and Circulating Variants of SARS-CoV-2 in Humans

As mentioned above, we identified 169 mutations in our system that also appeared in circulating strains in human populations, with normalized frequencies ranging from 0.142 to 610,131.60 per million SARS-CoV-2 genomes. These mutations were distributed in the 5′UTR, 3′UTR, and CDS of nine viral genes (Figure S4), reflecting a broad spectrum of prevalence and genomic localization.

Of the 28 convergent SNVs in our cellular experiments, many appeared sporadically in viral sequences in the spring of 2020 but did not persist in the predominant strains (Table S3 and Figures S4–S6). In contrast, two C>U mutations, C26681U in the *M* gene and C28311U in the *N* gene, demonstrated widespread prevalence (Fig. 6g). The C26681U mutation, observed in Calu-3 and CRFK cells, emerged in early January 2020 and quickly became a characteristic of the dominant viral strains. Similarly, the C28311U mutation, detected in Calu-3 and Vero E6 cells, appeared in early January 2020 and spread globally by February 2020. These observations indicate that the C>U mutational bias, potentially linked to APOBEC-mediated editing, could contribute to SARS-CoV-2 adaptability by supplying variation that may be subject to natural selection in circulating human strains.

Additionally, the A23040G|S:Q493R (detected in Huh-7, Caco-2, CRFK, and BHK-21 cells) and A23055G|S:Q498R (detected in CRFK and BHK-21 cells) mutations occur globally in the SARS-CoV-2 strains circulating in the human populations (Fig. 6g) and result in amino acid substitutions (Q493R and Q498R) that form additional salt bridges with ACE2 (Hong et al. 2022; Philip et al. 2023), strengthening the interaction (Fig. 6f). Both Q493R and Q498R mutations have been proposed as evidence for the mouse origin of Omicron (Wei et al. 2021), showed high prevalence after 30 passages in BHK-21 and CRFK cells. Their co-occurrence may facilitate viral replication in these otherwise nonpermissive cell lines (Table S3).

Together, these data demonstrate that our in vitro system not only captures key adaptive mutations relevant to interspecies transmission but also mirrors naturally occurring SARS-CoV-2 evolution in human populations. Moreover, the findings suggest that mutations enhancing cross-species transmission can readily arise and accumulate in nonhuman animals, underscoring the importance of strengthened viral surveillance in species closely associated with humans.

### IFN-I Modulates Mutation Patterns of SARS-CoV-2 in Cell Lines

**Fig. 7.**
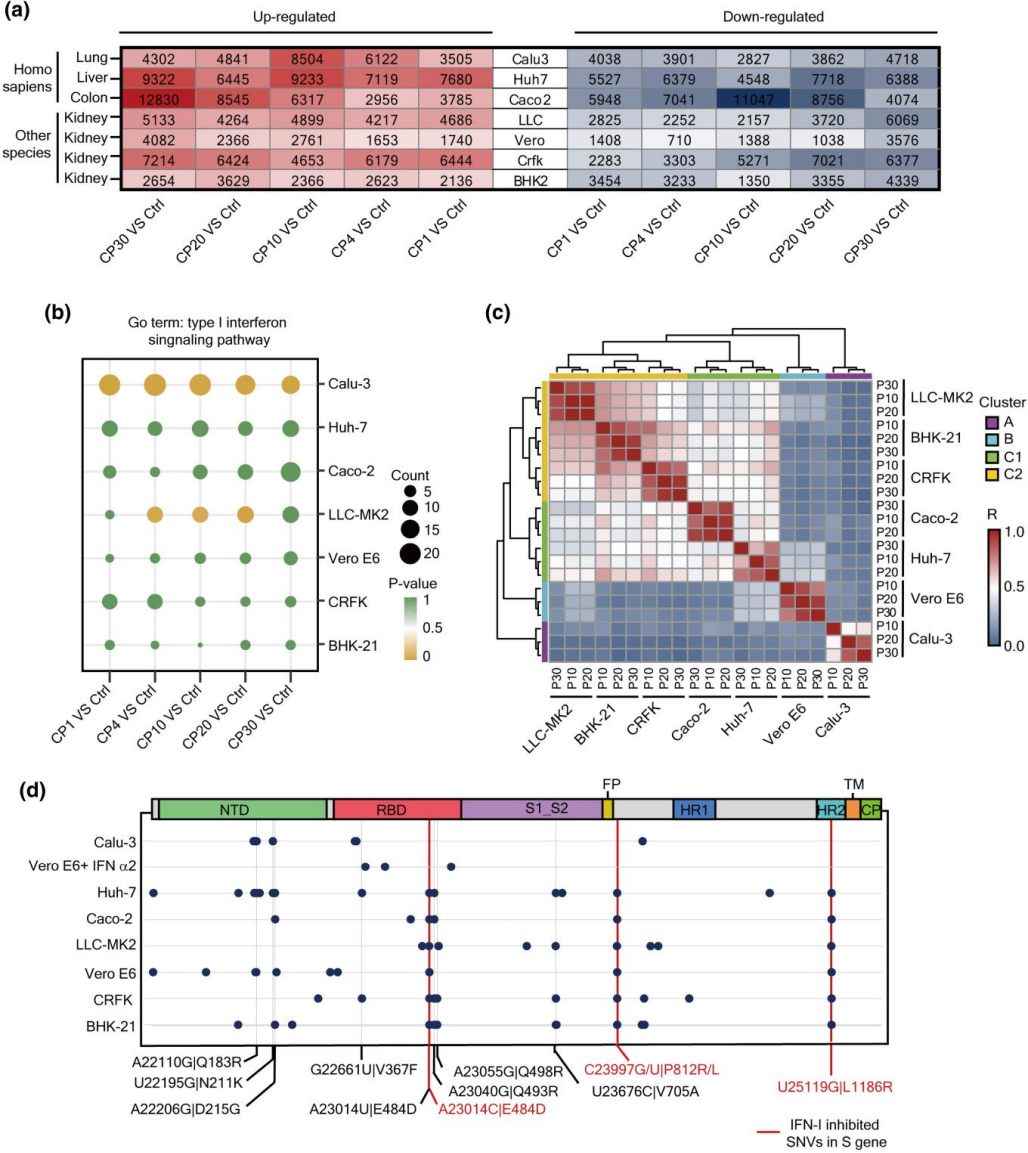
IFN-I signaling pathway inhibits the accumulation of E484D, P812R/L and L1186R mutations of SARS-CoV-2. a) Heatmap showing the number of upregulated (in red) or downregulated genes (in blue) for each passage relative to uninfected control of the indicated cell line. b) Bubble plot illustrating the differential enrichment of the IFN-I signaling pathway for the upregulated genes identified in each passage compared to the corresponding Ctrl in the indicated cell line. The function enrichment analysis was conducted by a webtool DAVID, with significance set at *P* < 0.05. The size of each bubble indicates the number of upregulated genes involved in the IFN-I signaling pathway. c) Heatmap showing the unsupervised hierarchical clustering of the *Pearson* correlation among the indicated cell lines with Calu-3 regarded as the root. The correlation has been calculated using the MuAF for all mutations identified at P10, P20, and P30 for all cell lines. d) Dot plot showing the convergent acquired mutations in *S* gene for all cell lines. After passing a patient-isolated SARS-CoV-2 in the indicated cell line for 30 passages, a series of acquired mutations have been convergently detected in *S* gene. The red lines indicate the mutations A23014C|S:E484D, C23997G/U|S:P812R/L, and U25119G|S:L1186R, which have been observed in all cell lines except for Calu-3 and Vero E6 + IFN α2 cells.

By analyzing sequencing data from cellular RNAs collected at the control (Ctrl) and subsequent passage points (CP1, CP4, CP10, CP20, and CP30) (Fig. 1b), we identified thousands of upregulated genes by comparing each time point of each cell line to the corresponding Ctrl (Fig. 7a). In particular, the functional enrichment analysis revealed that the IFN-I signaling pathway experienced the highest level of activation in Calu-3 cells (Fig. 7b and Figure S7a). These observations align well with the capacity of viral replication in it, highlighting the resistance factor that influences SARS-CoV-2's ability to surpass cell growth over time (Fig. 2c and d).

Consistent with the notion that the absence of IFN-I-encoding genes in Vero E6 cells (Geerling et al. 2022) and SARS-CoV-2 infection induces delayed and dysregulated IFN-I signaling in Huh-7 cells (Chen et al. 2021a), we found that no enrichment of genes in the significant activation of the IFN-I signaling pathway in these two cell lines. Besides, our results align with earlier studies indicating that neither SARS-CoV nor SARS-CoV-2 significantly triggered type I and type II interferon responses in Caco-2 cells (Shuai et al. 2020). Taken together, our RNA-Seq data suggested that IFN-I signaling was most activated in Calu-3 and was most suppressed in Vero E6 cells, with the other cell lines lying between these two cell lines (Fig. 7b and Figure S7a). These data suggest that the adaptability of SARS-CoV-2 in Calu-3 and Vero E6 cells differs markedly from the adaptability observed in other cell lines.

Heatmap analysis of the MuAF for all mutations at Passage 10, 20, and 30 identified three distinct clusters among the seven cell lines (Fig. 7c). The difference in occurrences and MuAF of mutations in different cell lines may result from varying intensities of selective immune pressure or other confounding factors across cell lines. Calu-3 forms Cluster A, whereas Vero E6 forms Cluster B, and the remaining five cell lines fall under Cluster C. Within Cluster C, Caco-2 and Huh-7 form subgroups designated Cluster C1, while the kidney cell lines such as LLC-MK2, CRFK, and BHK-21 from different species are grouped in Cluster C2 (Fig. 7c). These results showed that the strongest and weakest IFN-I signaling pathway can affect mutational landscapes of SARS-CoV-2. Our research showed that, unlike Calu-3 and Vero E6 cells, alterations in the IFN-I signaling pathway in other cell types do not have a significant effect on the adaptability of SARS-CoV-2 during passages, suggesting that the impact of IFN-I on viral evolution might be contingent on its concentration. Considering that Calu-3 cells exhibit robust IFN-I activation in response to SARS-CoV-2 (Park et al. 2021) and that Vero E6 cells lack genes associated with IFN-I pathway (Geerling et al. 2022), we proposed that the variations in MuAF among groups are influenced not only by tissue pressures but also by the innate immune response to the viral infection.

### IFN-I Signaling Pathway Restricts E484D Mutation Accumulation, Evidenced in Calu-3 Cells

Calu-3 cells exhibited higher activation of the IFN-I signaling pathway, potentially resulting in distinct mutation patterns of SARS-CoV-2 from other cell lines. First, mutations A23014C|E484D, C23997G|P812R/C23997U|P812L, and U25119G|L1186R in the *S* gene were identified in six other cell lines but were absent in Calu-3 cells (Fig. 7d). This finding also suggests that the IFN-I signaling pathway may restrict the accumulation of mutations E484D, P812R/L, and L1186R. To investigate whether strong activation of the IFN-I pathway in Calu-3 cells underlies this phenomenon, SARS-CoV-2 strain T was serially passaged in Vero E6 cells pretreated with increasing concentrations (1–10,000 IU/mL) of IFN-α2. Once viral resistance to IFN-α2 occurred, the supernatant RNAs of the virus variant (designated as Strain I) were collected for high-throughput sequencing. Given that the E484D, P812R/L, and L1186R mutations emerged in all other cell lines during passage experiments, except in Calu-3 cells (which display robust IFN-I activation), we hypothesized that these mutations would also arise in Strain I if the limiting effect of IFN-I is not involved. Sequencing revealed that E484D, P812R/L, and L1186R were absent in Strain I (Fig. 7d), supporting the notion that the IFN-I pathway suppresses the accumulation of these SARS-CoV-2 mutations. Additionally, the *ω* value of SNVs emerging in the *S* gene was higher overall than in other genes but was comparatively lower in Calu-3 cells than in other cell types (Fig. 4c). This pattern suggests that the *S* gene was subject to stronger selective pressure during passage in Calu-3 cells, potentially shaping a distinct mutational landscape in this cellular environment.

The E484D substitution has been shown to enhance SARS-CoV-2 infection in ACE2-negative cells by increasing TMEM106B binding (Baggen et al. 2023; Lacrampe and Hu 2023) and has been occasionally detected in circulating SARS-CoV-2 isolates (Figure S6). Intriguingly, compared to other host receptors of SARS-CoV-2, such as ACE2, ASGR1, KREMEN1, and TMEM106B exhibits broader and higher expression levels in both cell lines and tissues (Figure S7b and c), implying that TMEM106B may facilitate the infection of ACE2-negative cells in diverse tissues. These findings raise the prospect that TMEM106B-directed therapies, potentially in combination with interferon-based treatments, could be leveraged to limit viral spread—particularly under conditions of waning herd immunity. Indeed, a recent study demonstrated that a TMEM106B-specific monoclonal antibody (Ab09) effectively blocks SARS-CoV-2 infection in ACE2-deficient cells  (Baggen et al. 2023). However, given the essential role of TMEM106B in neurodevelopment  (Feng et al. 2021), we recommend confirming the presence of TMEM106B-dependent mutations (e.g. E484D) via viral genotyping prior to initiating TMEM106B-targeted therapies, to minimize off-target effects and safeguard host physiological function.

### IFN-I Showed Limited Effect on S Deletions During SARS-CoV-2's Adaptation to Cell Lines

Across the 36 samples, a total of 54 indels (including 50 deletions and 4 insertions) with a frequency greater than 0.01 in at least one sample were identified (Figure S8 and Table S4). Strikingly, all four insertions and 48 of the deletions were already detectable, albeit at very low levels, in the P0 inoculum, which had been initially isolated and amplified in Vero E6 cells. Consistent with previous reports that continuous passaging in Vero cells favors large deletions in S protein, particularly near the furin cleavage site (Q675_N679del and N679_A688del) and in the NTD (I68_T76del) (Funnell et al. 2021; Aiewsakun et al. 2023; Minami et al. 2024), we also detected these three deletions in the P0 population of the strain T, with initial frequencies of < 0.01, 0.01, and 0.01, respectively. During serial passaging, the I68_T76del rose steadily in frequency across all cell lines and eventually became fixed. In contrast, Q675_N679del reached fixation only in Vero E6 cells, persisted at intermediate frequency (∼0.2) in LLC-MK2 cells, and was lost in other cell types. Similarly, N679_A688del increased progressively in Vero E6, attaining a frequency of 0.34 by passage 30 but was eliminated in the other cell lines. These observations suggest that the I68_T76del is favored by all cell lines, and that Vero E6 cells, but not other cell systems, strongly favor the accumulation of S deletions near the furin cleavage site (Q675_N679del and N679_A688del).

To test whether this effect was attributable to the IFN-I deficiency of Vero E6 cells, we examined strain I, which had been continuously propagated in Vero E6 cells under IFN-α2 supplementation. In this strain, both I68_T76del and N679_A688del persisted, while Q675_N679del was absent. Thus, it is less likely that IFN-I strongly influences the accumulation of S deletions during Vero E6 passaging. Collectively, these findings indicate that IFN-I exerts only a limited influence on S deletions during SARS-CoV-2 adaptation. Mechanistically, this may reflect that large structural deletions in S are primarily constrained by protein stability and functional requirements, rather than immune-mediated pressures.

## Discussion

The ongoing evolution of SARS-CoV-2, marked by numerous mutations impacting disease severity and vaccine efficacy, underscores the importance of understanding viral adaptation. However, the limited genomic surveillance in humans and animals constrains our ability to monitor and predict emerging variants. Using a novel in vitro evolutionary system ensuring strict adherence to biosafety protocols, we explored SARS-CoV-2's adaptation in cell lines derived from diverse tissues and species, revealing significant insights into its convergent evolution. Our findings demonstrate that SARS-CoV-2 undergoes strong positive selection in the S protein in all cell lines, highlighting its adaptability even in the absence of adaptive immune or therapeutic pressures. This adaptability is reflected in increased CPEs and replication efficiency, with the exception of Calu-3 cells, where robust innate immune responses, particularly IFN-I, appeared to mitigate viral adaptation. Additionally, we observed an accumulation of C>U mutations during serial passages, potentially influenced by APOBEC enzymes, aligning with the predominance of C>U mutations in SARS-CoV-2 circulating within human populations (Di Giorgio et al. 2020; Ratcliff and Simmonds 2021). The dataset we generated provides a unique framework for dissecting host-specific evolutionary trajectories. While a large number of mutations were unique to individual cell types, only 28 sites exhibited convergent evolution in at least two, underscoring the influence of cell types-specific selective pressures. These patterns likely reflect differences in IFN-I signaling, species origin, and tissue derivation, each of which may shape the intensity and direction of selective pressures. As shown in Fig. 7c, heatmap analysis of MuAF revealed three major clusters in the seven cell lines. Calu-3 cells, which cluster in group A, mount a robust IFN-I response that limits the accumulation of mutations such as E484D, P812R/L, and L1186R. In contrast, Vero E6 cells (cluster B), which lack functional IFN-I genes, provide a permissive environment for viral replication and diversification. While IFN-I signaling is a major determinant, mutation profiles also varied by species and tissue origin. For instance, LLC-MK2, CRFK, and BHK-21 cells—despite having only moderate IFN-I activity—exhibited distinct mutational signatures compared to human-derived lines such as Caco-2 and Huh-7. Clustering analysis grouped Caco-2 and Huh-7 into cluster C1, whereas kidney-derived lines from multiple species (LLC-MK2, CRFK, and BHK-21) were assigned to cluster C2, suggesting that tissue origin exerts an additional layer of selective constraint beyond innate immunity. Together, these findings highlight the multifactorial nature of SARS-CoV-2 adaptation in vitro and reinforce the importance of host cell context—both immunological and anatomical—in shaping viral evolution.

Of the convergent mutations identified through in vitro evolution, only four (M:F53F, N:P13L, S:Q493R, and S:Q498R) reached appreciable prevalence in human populations (Fig. 6g), highlighting a disconnect between cell-based mutational dynamics and those shaped by natural transmission. Several factors may account for this discrepancy. First, in vitro systems lack the full spectrum of host immune components, particularly adaptive responses, which exert strong selective pressure in vivo and can drive immune escape mutations. Second, mutations that enhance replication in isolated cell lines may incur fitness costs in the context of whole-organism infection, where viruses must balance replication efficiency with transmissibility and immune escape (Carabelli et al. 2023). Third, intra-host and inter-host selective pressures are often misaligned: while diverse variants may arise within individuals, few are successfully transmitted across hosts due to evolutionary bottlenecks and tradeoffs  (Hou et al. 2023; Manrique et al. 2024; Tai et al. 2025). Fourth, context-dependent epistasis and pleiotropy influence which mutations are retained (Tang et al. 2021; Tai et al. 2025); for instance, Q498R exhibits positive epistasis with N501Y, enhancing ACE2 binding only when co-occurring (Moulana et al. 2022; Starr et al. 2022). Additionally, mutation effects may vary across host species or tissue types, such that mutations conferring a fitness advantage in kidney-derived or nonhuman cells fail to translate to human respiratory epithelium. The genetic background of the viral isolates may also shape evolutionary trajectories, with certain mutations emerging preferentially in specific genomic contexts. Finally, population-level factors such as vaccination, behavioral interventions, and stochastic effects further influence which mutations reach fixation. Together, these observations underscore the value of in vitro systems for mapping mutational potential while emphasizing the need to contextualize findings within the complex landscape of host biology and real-world transmission dynamics.

Viral infection triggers the activation of IFN-I signaling, which leads to the induction of hundreds of ISGs that function collectively to inhibit viral replication (Park and Iwasaki 2020). This antiviral program imposes substantial selective pressure on the virus, shaping its evolutionary trajectory. In Calu-3 cells, we observed sustained expression of ISGs such as ISG15, OAS2, RNASEL, and MX1, which act through distinct antiviral mechanisms: ISG15 interferes with N protein dimerization to block virion assembly  (Bang et al. 2024); OAS2 activates RNASEL, leading to degradation of viral RNA and inhibition of protein synthesis (Li et al. 2021); and MX1 interacts with the viral RNA-dependent RNA polymerase (RdRp) to reduce replication efficiency (Bizzotto et al. 2020). This sustained antiviral state likely lowers viral replication efficiency and thereby reduces the probability of de novo mutations arising during genome replication. Despite this restrictive environment, SARS-CoV-2 may still evolve mutations that counteract ISG-mediated inhibition or reduce dependency on host factors. For instance, mutations such as E484D and P812R in the S protein have been implicated in modulating receptor usage or immune evasion (Dieterle et al. 2020; Baggen et al. 2023; Vizgirda et al. 2025). However, these mutations did not emerge in Calu-3 cells, suggesting that their associated fitness cost in a high-IFN context outweighs any potential selective advantage. Mechanistically, it is also possible that strong ISG-mediated immune pressure directly suppresses the occurrence or segregations of certain mutations such as E484D and P812R. This hypothesis is supported by our observation that pretreatment of Vero E6 cells with IFN-α2 significantly suppressed the emergence of E484D, P812R/L, and L1186R, demonstrating that IFN-I signaling acts as a potent selective barrier against specific adaptive mutations. In contrast, cell lines with weak (Huh-7, Caco-2, Vero E6, and BHK-21) or declining (LLC-MK2 and CRFK) IFN-I signaling exhibited higher viral replication rates and allowed greater accumulation of mutations, including E484D and P812R. These environments represent permissive evolutionary landscapes, where the virus experiences less immune pressure and can tolerate a broader range of mutations. Collectively, these findings highlight a complex interplay between immune pressure and the viral mutational landscape, underscoring the need for further studies to elucidate the mechanisms that govern this relationship.

In this study, we identified 28 SNVs that emerged in at least two distinct cell lines, suggesting the presence of convergent evolution and potential mutational hotspots or adaptive constraints favoring specific genomic changes in vitro. To further explore these patterns, we compared our dataset with two prior studies that investigated SARS-CoV-2 adaptation in cell culture: Chen et al. (2021b) and Ramirez et al. (2021). Both studies employed similar host systems, including human-derived Huh-7/Huh-7.5 cells and primate-derived Vero E6 cells, paralleling our own experimental setup. Interestingly, no convergent mutations were observed between Chen's dataset and either ours or Ramirez's, highlighting the substantial divergence in independent passaging experiments. In contrast, several shared mutations were observed when comparing our findings to those of Ramirez et al. In Vero E6 cells, three of the 25 mutations reported by Ramirez—U11522G|ORF1ab:F3753V, A23014C|S:E484D, and C26333U|E:T30I—were also detected in our strains. Notably, these same three mutations also appeared in both our and Ramirez's Huh-7/Huh-7.5-derived strains, and an additional mutation, C23997G|S:P812R, was shared between Ramirez's Huh-7.5 and our Huh-7 strains. These overlapping mutations likely reflect common adaptive responses to shared cellular environments or passage-related pressures. The divergence between studies, despite similarities in cell lines, may be attributed to differences in the initial viral inocula, culture protocols, and the number of passages (Table S5), consistent with findings in mouse-adapted strains. The repeated fixation of F3753V, E484D, and T30I in both Vero and Huh-derived strains in our and Ramirez's studies strongly supports the notion of cell-type-driven convergent adaptation. We also identified 54 indels across 36 samples during serial passaging (Figure S8). Consistent with previous reports (Funnell et al. 2021; Aiewsakun et al. 2023; Minami et al. 2024), deletions in the S protein—particularly near the Furin cleavage site and within the NTD—accumulated during propagation in Vero E6 cells. Specifically, I68_T76del became fixed in all cell lines, while Q675_N679del and N679_A688del were predominantly maintained in Vero E6 cells but diminished in other cell lines by passage 30. The initial isolation and amplification of strain T (P0 virus stock) were performed in Vero E6 cells, and these deletions, present at low frequencies in the original stock, were differentially enriched during passaging in cell types. Together, these results underscore the influence of host cell environment in shaping both SNV and indel dynamics during the in vitro evolution of SARS-CoV-2.

Our cellular experiments demonstrate that SARS-CoV-2 can rapidly adapt to different cell types, leading to enhanced CPEs or increased replication efficiency. Although CPE provides a useful indicator of virus-induced cellular damage such as cell swelling, fusion, apoptosis, and lysis—these changes do not necessarily indicate that SARS-CoV-2 exhibits a similar trend in the human population. This is because CPE is measured at the cellular level, while viral virulence in human populations is determined at the host level and influenced by complex interactions among the pathogen, host immune responses, and environmental factors (Casadevall and Pirofski 2001). Thus, changes in CPE observed in vitro do not always correlate with pathogenicity at the organismal level. Recent studies of SARS-CoV-2 variants illustrate this disconnection: both Delta and Omicron variants exhibit reduced CPE in commonly used cell lines compared to ancestral strains (Phadke et al. 2024), yet Delta is associated with increased pathogenicity in humans and animal models, whereas Omicron displays lower virulence (Bager et al. 2022; Halfmann et al. 2022; Saito et al. 2022; Suzuki et al. 2022; Wolter et al. 2022). This divergence is likely shaped by variant-specific mutations affecting viral entry and fusion (e.g. the P681R substitution in Delta) and by altered tissue tropism and fusogenicity in Omicron (Aggarwal et al. 2022; Hui et al. 2022; Saito et al. 2022; Suzuki et al. 2022). Given that our study is based on in vitro systems, we are unable to directly assess virulence or transmissibility in vivo. Nonetheless, our findings underscore the importance of integrating both cellular and organismal data to fully elucidate the consequences of viral evolution and adaptation.

Because this study is a longitudinal passaging experiment, excluding low-frequency variants would bias against the earliest stages of adaptation: nascent beneficial mutations typically arise and remain rare before expanding. We therefore retained low-frequency calls (MuAF ≥ 0.01) to monitor evolutionary trajectories while guarding against artifacts via high depth (≥ 100 reads/site), a temporal-consistency requirement (appearance across ≥ 4 passages for “acquired” variants). Variants with MuAF < 0.01 were omitted because, although some may represent true mutations, they are more likely to reflect sequencing artifacts. Focusing on MuAF ≥ 0.01 is justified, as analyses using a stricter threshold (≥ 0.05; Figure S1) produced consistent results, including positive selection in the S protein and APOBEC-mediated C > U editing, supporting the robustness of our conclusions. To account for host-specific permissivity, MOIs were optimized per cell line (Table S1). While variable MOIs can influence bottleneck severity and defective particle accumulation, our main observations were consistent across conditions: (i) convergent mutations (e.g. S:E484D, S:P812R/L) arose in both high- and low-MOI lines; (ii) host-related factors (e.g. IFN-I activity, tissue origin) explained mutational clustering more strongly than MOI; and (iii) APOBEC-mediated C>U bias increased universally. Nevertheless, MOI heterogeneity remains a limitation for cross-line comparisons of evolutionary rates, and future work may benefit from ddPCR, single-cell sequencing, or adjusted passaging protocols to minimize this confounder.

In summary, our study demonstrates the remarkable adaptability of SARS-CoV-2 to various host environments and highlights the importance of using a diverse array of cell lines to study viral evolution. The rapid adaptation of the virus to different cell cultures, accompanied by increased CPEs and replication efficiency, underscores the need for continued surveillance and research to understand the evolutionary dynamics of SARS-CoV-2 and develop effective strategies to combat its spread. By examining the role of APOBECs and IFN-I in shaping SARS-CoV-2's evolution, we demonstrate that the intracellular environment of the host can act as a critical selective pressure, driving the emergence of viral adaptations that enhance immune evasion and transmission. This finding underscores the need for a more holistic approach to studying viral evolution, one that considers the complex interplay between viruses and their hosts.

## Materials and Methods

### Isolation of SARS-CoV-2

The SARS-CoV-2 (strain T), used in this study, was isolated from the sputum sample of an imported case. This patient returned to China from Yangon, Myanmar in September 2020, and was hospitalized and quarantined at Yunnan Infectious Diseases Hospital for treatment. Immediately after collection, the sputum sample was transported to the BSL-3 of Kunming Institute of Zoology, CAS on ice in accordance with relevant biosafety management regulations and used for SARS-CoV-2 isolation. The sample was resuspended in five times volume medium (dulbecco's modified eagle medium (DMEM) with 2% fetal bovine serum (FBS), 100 U/mL ampicillin, 100 μg/mL streptomycin, and 2.5 μg/mL amphotericin B) (Thermo Fisher, USA). Then, 2 mL supernatant was cocultured with Vero E6 cell in a T25 flask for 2 h, followed three times washing with phosphate buffered saline (PBS). After 5 days of culture in the same medium, the culture supernatant was collected and filtered with a 0.22 μm filter. Then, they were added to the T75 flask to infect Vero E6 cells for 2 h, followed by three times washing with PBS. After 3 days' culture in maintained medium (DMEM) with 2% FBS, 100 U/mL ampicillin, and 100 μg/mL streptomycin), the culture supernatant was collected, filtered, aliquoted, and titrated for use.

### Serial Passage of SARS-CoV-2

Cells of Calu-3, Caco-2, Huh-7, Vero E6, LLC-MK2, CRFK, and BHK-21 were cultured with complete medium (DMEM with 10% FBS, 100 U/mL ampicillin, and 100 μg/mL streptomycin). When the cells reached 85–95% confluent monolayer, they performed 1:3 passaging with 0.25% trypsin digestion. In a six-well culture plate, 4.0 × 10^5^ cells of Caco-2, Huh-7, Vero E6, LLC-MK2, CRFK, and BHK-21 were seeded with 2 mL complete medium, while 1.5 × 10^5^ Calu-3 cells were seeded in a 12-well culture plate with 500 μL complete medium. There were six duplicate wells used in this study for each type of cell. Subsequently, the cells including Calu-3, Caco-2, Huh-7, Vero E6, LLC-MK2, CRFK, and BHK-21, were infected with SARS-CoV-2 at different MOI for 2 h in a maintenance medium during the initial stage of infection: 0.05, 0.05, 0.2, 0.01, 0.4, 0.4, and 0.4, respectively. The MOI used for each cell line is to balance viral load, CPE progression, and serial propagation viability according to the preliminary experiments (data not shown). Then, mediums were replaced with the maintenance medium. After 3 days, the supernatant was collected by centrifugation, then aliquoted, and stored frozen for future use or promptly utilized for subculturing. Trizol reagent (Thermo Fisher, USA) was applied to the culture plate and cell vial for 15 min, after which the samples were collected and stored at −80 °C for RNA extraction. To achieve successful serial passage of the virus, supernatants from Calu-3, Caco-2, Huh-7, and Vero E6 were individually diluted 2, 10, 10, and 200 times, respectively, using maintenance medium for the subsequent infection rounds. Conversely, the supernatants from LLC-MK2, CRFK, and BHK-21 were used directly without dilution. After an infection of 2 h, the growth medium was replaced with a maintenance medium. The cells were then cultured for 3 days, after which the supernatant was collected for the next round of infection. Supernatant and cell lysate were collected and frozen after each passage, to be used up to the 30th generation. All experiments and subsequent analyses involving SARS-CoV-2 were conducted under strict biosafety management in the BSL-3 at the Kunming Institute of Zoology, Chinese Academy of Sciences. The experimental period of this study spans from May 2022 to March 2023. After the research is completed, all samples were subjected to high-pressure and high-temperature destruction.

### Cytopathic Effect Assays

Observations of cellular pathology, such as rounding, detachment, wrinkling, rupture, and cell death, were conducted before harvesting the supernatant at each passage. Cellular pathology was categorized into four levels based on the degree of cell changes: grade I (less than 25% of cells show pathological alterations), grade II (more than 25% but less than 50% of cells exhibit pathological changes), grade III (more than 50% but less than 75% of cells display pathological changes), and grade IV (more than 75% of cells demonstrate pathological changes).

### Comparing the Replication and Pathogenicity of Viruses

The viral titers of P0 and P30 were determined on Vero E6 cells. Subsequently, equal MOIs of P0 and P30 virus were used to infect Calu-3, Caco-2, Huh-7, Vero E6, LLC-MK2, CRFK, and BHK-21 cells, with MOIs of 0.5, 0.5, 0.5, 0.01, 0.5, 0.5, and 0.5, respectively. Following a virus incubation period of 2 h, cells were washed with PBS 3 times and then incubated for 72 h. Cell morphology was observed under a microscope, and photos were taken at 72 h postinfection. Half of the culture medium was collected for viral load detection, while an equal volume of maintenance medium was added to the culture plate. The viral load of culture supernatant was performed by quantitative PCR as previously described (Tian et al. 2021). Briefly, nucleic acid was extracted using the High Pure Viral RNA Kit (Roche, Germany). Then, SARS-CoV-2 *N* gene was detected using a Probe One-Step qRT-PCR Kit (Toyobo, Japan) and *N* gene-specific primer and probe (Table S6). Amplification was performed using QuantStudio5 (Applied Biosystems, Thermo Fisher, USA). The copies of the *N* gene were calculated by QuantStudio Design & analysis software (v1.5.1) based on SARS-CoV-2 RNA reference material (National Institute of Metrology, China, GBW(E)091089).

### Next-generation Sequencing

The supernatant from six replicated wells of identical cell types was pooled in equal amounts for nucleic acid extraction using the High Pure Viral RNA Kit. Nucleic acids obtained from P0, P1, P4, P10, P20, and P30 were subjected to next-generation sequencing using DNBseq (100 bp paired-end reads were generated, BGI Genomics). Because viral supernatant RNA lacks 18S/28S rRNA and contains carrier RNA from the High Pure Viral RNA Kit, standard Bioanalyzer RIN and NanoDrop measurements were not applicable. Instead, RNA samples with concentrations ≥ 10 ng/µL (Qubit 4.0, Thermo Fisher) were used for library preparation. This concentration-based QC criterion ensured reproducibility across samples and was validated by consistent downstream sequencing quality metrics. Nucleic acids were fragmented for cDNA synthesis with adaptor ligation. Libraries were generated through PCR amplification. Qualifying products were denatured and circularized to form single-stranded circular DNA molecules, which were replicated via rolling cycle amplification to create DNA nanoballs (DNBs) containing multiple DNA copies. High-quality DNBs were loaded onto patterned nanoarrays using the high-intensity DNA nanochip technique and sequenced through combinatorial Probe-Anchor Synthesis (cPAS).

The total cellular RNAs from uninfected control, CP1, CP4, CP10, CP20, and CP30 were used for RNA-Seq. Briefly, equal volumes of cell lysate from six repeated wells of identical cells were combined. The RNA was subjected to mRNA enrichment using a Dynabeads^TM^ mRNA purification kit (Thermo Fisher Scientific, USA). Fragmentation was carried out using divalent cations under elevated temperature in NEBNext First Strand Synthesis Reaction Buffer (5×) (New England Biolabs, USA). Subsequently, fragmented RNAs were subjected to first- and second-strand cDNA synthesis, followed by adaptor ligation and enrichment using NEBNext Ultra RNA Library Prep Kit for Illumina (New England Biolabs, USA). The purified library products were evaluated using Qubit (v2.0; Life Technologies, USA) and Agilent 2100 bioanalyzer (Agilent, USA). The library preparations were sequenced on an Illumina Novaseq 6000 platform (Novogene, USA), and 150 bp paired-end reads were generated.

### Sequencing Data Processing

Firstly, adapter contamination was identified and removed using Cutadapt v1.18 (Martin 2011). Then, low-quality RNA sequencing (RNA-seq) reads were filtered using Trimmomatic v0.39 (Bolger et al. 2014). For each supernatant sample, the filtered reads were, respectively, mapped to the SARS-CoV-2 reference genome by BWA v0.7.17 (Li and Durbin 2009). For SNV calling, only uniquely mapped reads with a mapping quality score ≥ 30 were retained to ensure high-confidence alignments. SNVs were identified using Freebayes v1.3.6 (Garrison and Marth 2012), with the following parameters: −haplotype-length 0 to disable haplotype-based calling, -m 30 to require a minimum base quality of 30 for supporting evidence, -q 20 to exclude reads with low base quality scores, −ploidy 1 to reflect the haploid nature of the viral genome, and -F 0.01 to set a minimum variant allele frequency threshold of 1%. The average sequencing depth across the SARS-CoV-2 genome was 120,688.30 reads per nucleotide for each sequencing dataset. Applying a depth threshold of ≥ 100 allowed us to cover 99.8% of the viral genome in each dataset, thereby ensuring sufficient coverage for accurately distinguishing true variants from potential sequencing errors. To reduce false positives while balancing sensitivity and specificity, we filtered SNVs with ≥ 100 reads and MuAF ≥ 0.01. For higher confidence, we also applied a stricter filter with MuAF ≥ 0.05.

We employed a two-step procedure to estimate the frequency of insertion–deletion mutations (indels) in SARS-CoV-2 in samples. First, candidate indels were identified using Freebayes v1.3.6. To validate and accurately quantify their frequencies, we directly searched the raw sequencing reads for evidence supporting each indel by comparing alignments to the sequences before and after the indel event. Specifically, for each candidate indel, we extracted 200 nucleotides upstream and 200 nucleotides downstream from the reference genome to construct a sequence fragment (termed the “reference sequence”). In parallel, we generated a corresponding “mutant sequence” reflecting the indel event. We then aligned all raw sequencing reads to both the reference and mutant sequences using BLAT  (Kent 2002) and determined the best match for each read. We only considered the NGS reads that spanned the indels. The frequency of each indel was then estimated as the proportion of reads that aligned more accurately to the mutant sequence relative to the total aligned reads. Indels with a frequency greater than 0.01 in at least one sample were retained for downstream analysis.

For cellular mRNAs, the remaining reads were, respectively, aligned to the corresponding genome (*Homo sapiens*, *Chlorocebus sabaeus*, *Macaca mulatta*, *Mesocricetus auratus* or *Felis catus*) augmented with Ensembl (release 109) genome annotation using HISAT2 (Kim et al. 2019). To minimize the rate of false positives, only uniquely mapped reads with -q ≥ 20 were kept for the following analysis for each sample. The counts matrix was then obtained using HTSeq (Putri et al. 2022), with parameters “−mode = union and −stranded = no’. Differential gene expression (DEG) analysis was conducted by Bioconductor package DEGSeq (Wang et al. 2010). Genes with |log_2_ (Fold change)| > 1 and FDR < 0.05 were considered as significant. Function enrichment for DEGs was conducted by DAVID webtools (https://davidbioinformatics.nih.gov/). Only terms with *P* < 0.05 were regarded as significant.

### Ka/Ks Calculation

For each cell line, we separately calculated the per-passage Ka/Ks (Fig. 3; SNVs arising relative to P0), and the Ka/Ks of acquired SNVs (Fig. 4c). The Ka and Ks were computed according to the formulas Ka = (nonsynonymous substitutions)/(nonsynonymous substitutions) and Ks = (synonymous substitutions)/(synonymous substitutions). The numbers of synonymous and nonsynonymous sites for the concatenated all other gene CDSs and *S* gene of the reference genome were obtained by YN00 from PAML v4.9a (Li et al. 1985; Nei and Gojobori 1986; Yang 2007).

### Transmission Analysis of Viral SNVs

According to the transmission chain, viral SNVs were categorized as extinct, stable, and acquired. Taking the transmission analysis in Calu-3 cells as an example, the process to determine the occurrence and development of SNVs is outlined as follows:

1. Only SNVs with sequencing depth ≥ 100 in all passages have been kept.
2. The SNVs in the incomplete transmission chain were discarded. For example, if the same variation site was detected in P0, P1, P10, P20, P30, except for P4, it would be regarded as an incomplete transmission chain (because it is not certain whether this variation disappears in P4 or does not meet the criteria of SNV calling).
3. SNVs detected in P0 but not present in subsequent passages were identified as extinct variants. SNVs found in P0 and transmitted through all passages were labeled as stable variants. SNVs discovered in P1 and passed down in the following passages were identified as acquired variants.

### Analysis Tools

The sequence logos were created by WebLogo 3 online tool with probability units https://weblogo.threeplusone.com/). The lineage analysis of SARS-CoV-2 (strain T) was conducted by Nextstrain online tool (https://clades.nextstrain.org/). The frequency of the indicated variants in the population was calculated using the epicov dataset as of Jun 5, 2025, sourced from Global Initiative on Sharing All Influenza Data (GISAID, https://gisaid.org/).

## Supplementary Material

msaf274_Supplementary_Data

## Data Availability

The RNA-seq of this study data have been deposited into National Genomics Data Center (https://ngdc.cncb.ac.cn/gsa-human) with accession number: HRA007667.

## Code Availability

The main code has been deposited in github under https://github.com/Candiesbio75/Convergent-evolution-of-SARS-CoV-2. Source codes are available from the authors upon request.
