# Supplementary material for: Convergent Evolution and Host-Limiting Impacts of SARS-CoV-2 Revealed by Cellular Experiments
Source: Mol Biol Evol. 2025 Oct 28;42(11):msaf274. doi: 10.1093/molbev/msaf274 (PMC12619125; doi:10.1093/molbev/msaf274)
Supplement: msaf274_Supplementary_Data [file msaf274_supplementary_data.zip › Supplementary Information with figures and tables (MBE-25-0068).docx]

**Supplementary Materials**

**Convergent evolution and host-limiting impacts of SARS-CoV-2 revealed by cellular experiments**

Ting Zhang^1,6^, Ren-Rong Tian^1,6^, Fengyi Li^2,3,6^, Xiaolu Tang^4,6^, Wenbin He^2^, Zhen-Ping Hao^1^, Lin Zhuo^1,3^, Jian Lu^4, 5,*^, Xuemei Lu^2,3,*^, Yong-Tang Zheng^1,*^

^1^State Key Laboratory of Genetic Evolution & Animal Models, Key Laboratory of Bioactive Peptides of Yunnan Province, KIZ-CUHK Joint Laboratory of Bioresources and Molecular Research in Common Diseases, Center for Biosafety Mega-Science, Kunming Institute of Zoology, Chinese Academy of Sciences, Kunming, China.

^2^State Key Laboratory of Genetic Evolution & Animal Models, Yunnan Key Laboratory of Biodiversity Information, Kunming Institute of Zoology, Chinese Academy of Sciences, Kunming, Yunnan, China.

^3^University of Chinese Academy of Sciences, Beijing, China.

^4^State Key Laboratory of Gene Function and Modulation Research, Center for Bioinformatics, School of Life Sciences, Peking University, Beijing, China.

^5^Beijing Advanced Center of RNA Biology (BEACON), Peking University, Beijing, China.

^6^These authors contributed equally: Ting Zhang, Ren-Rong Tian, Feng-Yi Li, Xiao-Lu Tang

*Corresponding author:

Yong-Tang Zheng: zhengyt@mail.kiz.ac.cn

Xuemei Lu: xuemeilu@mail.kiz.ac.cn

Jian Lu: luj@pku.edu.cn

**
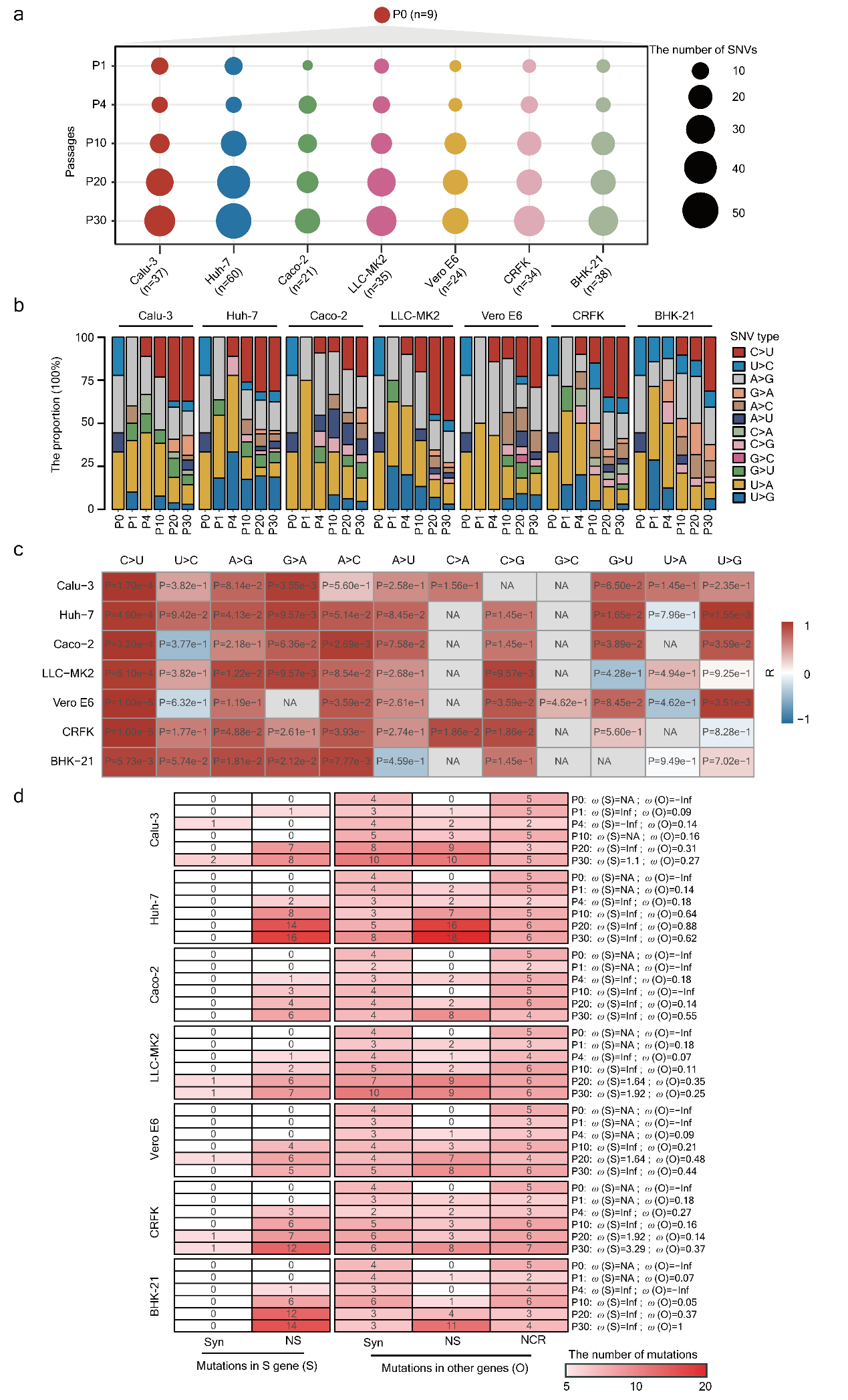
**

**Fig. S1. The number of SARS-CoV-2 SNVs identified in the indicated cell lines during passages. a**, Bubble plot illustrating the number of SARS-CoV-2’s SNVs identified during the indicated passage in the indicated cell line. The size of each bubble represents the number of SNVs identified, with larger bubbles indicating more SNVs. N represents the total number of SNVs identified in all passages for each cell line after deduplication. **b**, Bar plots showing the frequency of twelve mutation types of SARS-CoV-2 in each passage in the indicated cell line. **c**, Heatmap depicting the relationship between the normalized ratios of each mutation type of SARS-CoV-2 and the passages. R denotes the *Pearson* correlation coefficient for the indicated character and the passages, with the statistical significance assessed using *Pearson*’s test. **d**, The *ω* values calculated for *S* gene (S) and other genes (O). The number of mutations has been shown on the heatmap. The threshold used for SNV identification is sequencing depth ≥ 100 and MuAF ≥ 0.05.

**
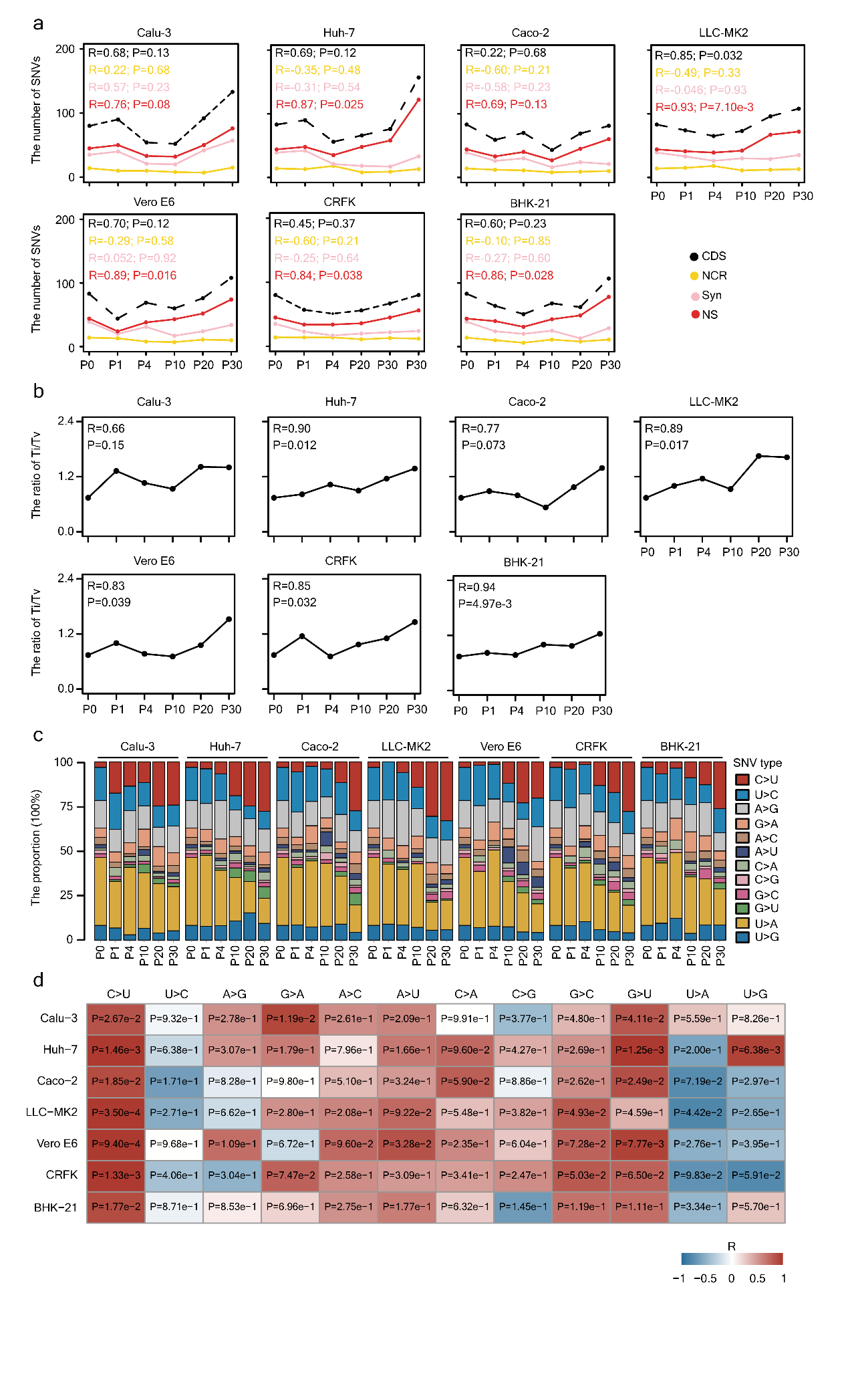
**

**Fig. S2. Characteristics of the identified SNVs along the SARS-CoV-2 genome. a**, Line plots showing the temporal trend of the number of SNVs detected in CDS (in black) and NCR (in gold). Moreover, the SNVs distributed in CDS have been grouped into Syn (in pink) or NS (in red). **b**, Line plots display the temporal increase of the Ti/Tv in each cell line. **c**, Bar plots showing the frequency of twelve mutation types of SARS-CoV-2 in each passage in the indicated cell line. **d**, Heatmap depicting the relationship between the normalized ratios of each mutation type of SARS-CoV-2 and the passages. In a, b and d, R denotes the *Pearson* correlation coefficient for the indicated character and the passages, with the statistical significance assessed using *Pearson*’s test.

**
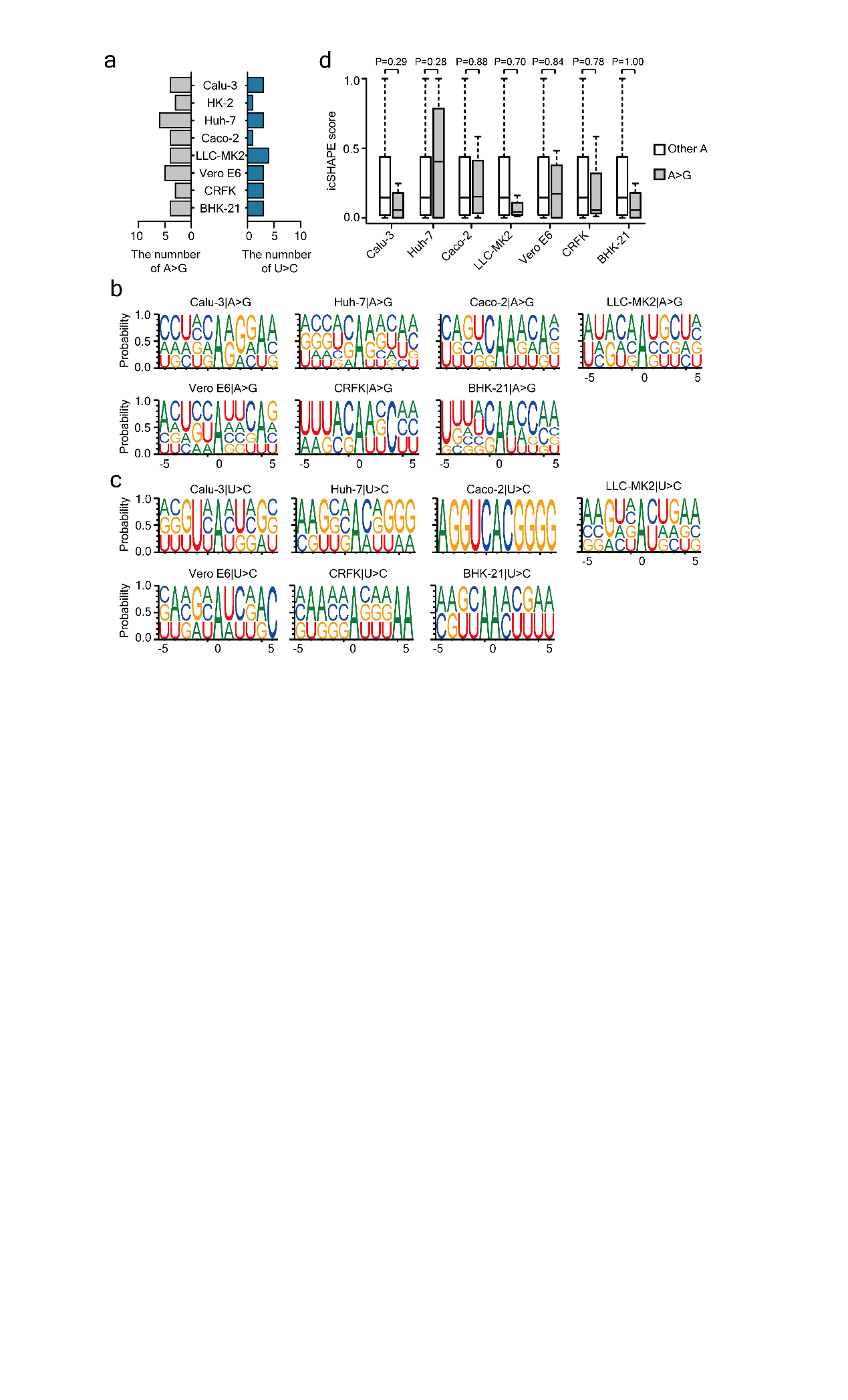
**

**Fig. S3. ADAR shows less influence on the formation of A>G and U>C mutations. a**, Bar plot showing the number of acquired A>G (in grey) or acquired U>C (in blue) SNVs of SARS-CoV-2. **b**, **c**, Sequence motifs of the acquired (b) A>G or (c) U>C SNVs of SARS-CoV-2 with flanking ± 5 nucleotides in the indicated cell line. For U>C, the reverse complementary sequences have been used. **d**, Distribution of the *in vivo* icSHAPE reactivity scores at the A sites, with (in grey) or without (in white) acquired A>G mutations in the indicated cell line. The statistical significance was calculated by unpaired one-tailed Kolmogorov-Smirnov test.

**
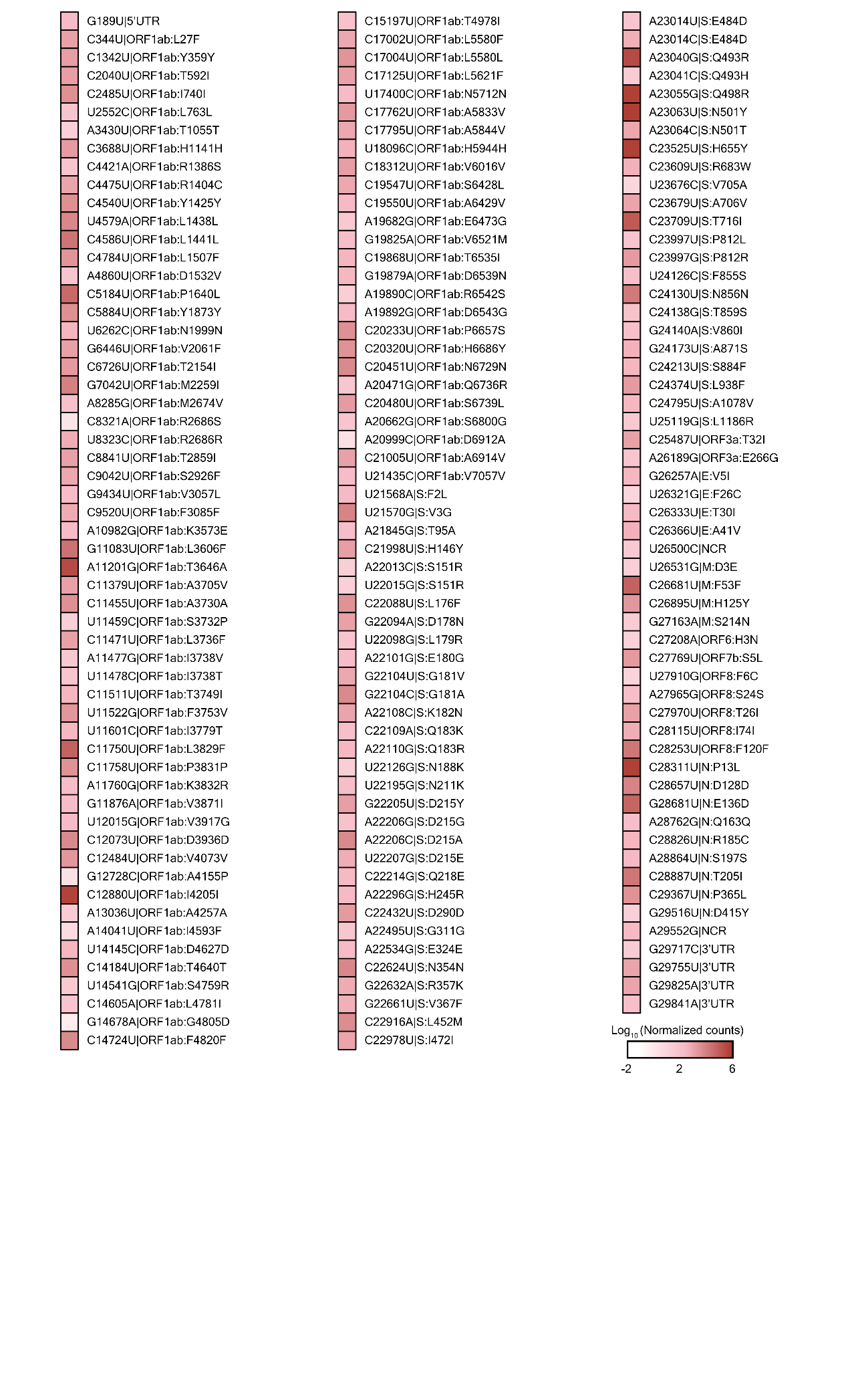
**

**Fig. S4.** **Comparison of acquired mutations from *in vitro* viral passage with SARS-CoV-2 strains circulating in human populations.** A total of 169 acquired mutation sites identified in our experimental system were also detected in global SARS-CoV-2 genomic data, with normalized counts ranging from 0.142 to 610131.6 per million sequences. These mutations were distributed in the 5’UTR, 3’UTR, and CDS of nine viral genes. Mutation frequencies were calculated using sequence data retrieved from the GISAID database (accessed on Jun 5, 2025). After quality control, 13,988,408 genomes were used for further normalization analysis. The quality control criteria included: 1) Ns < 5%; and 2) length > 29,000 after removing Ns.

**
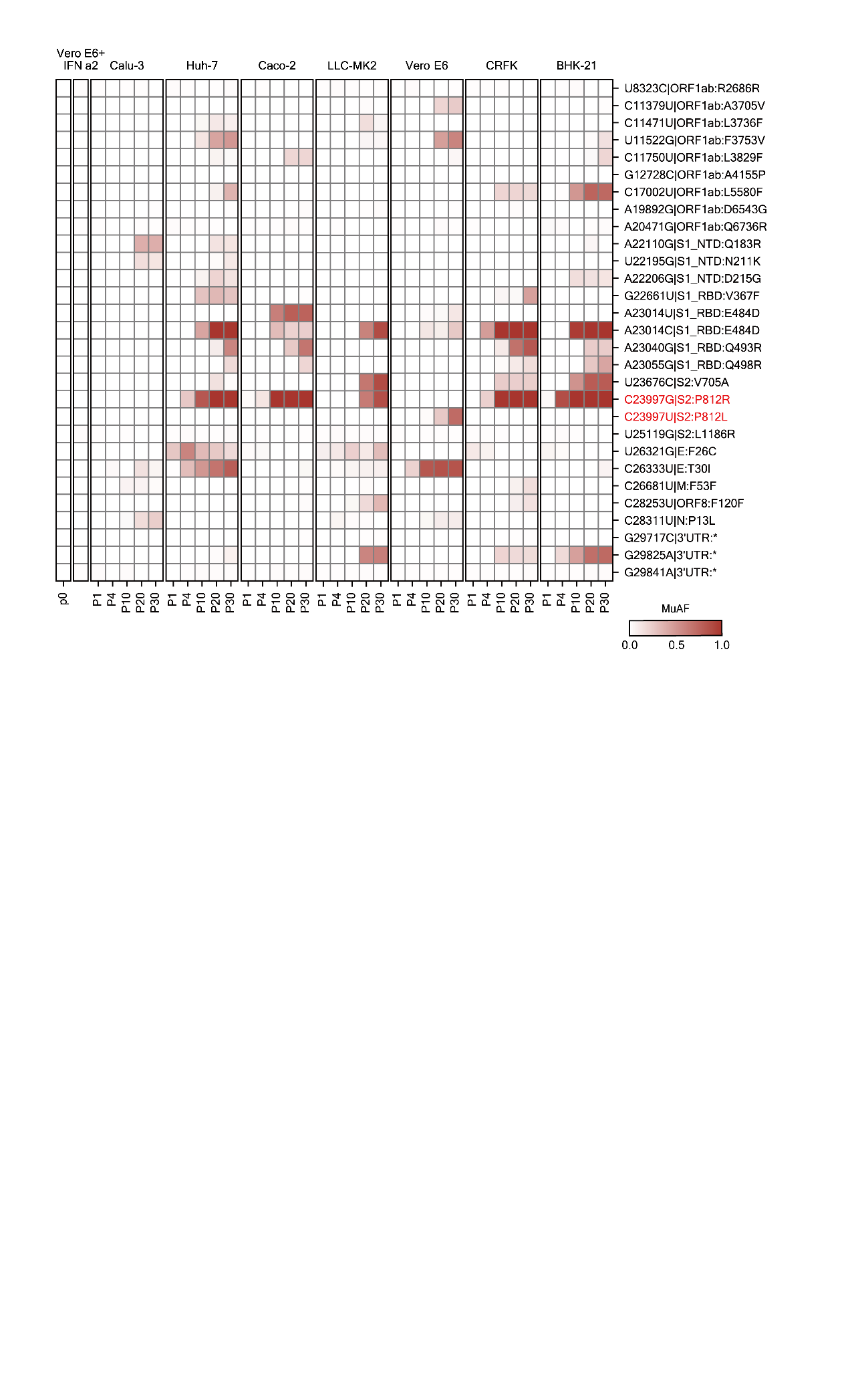
Fig. S5. The MuAF level for 28 convergent SNVs for each cell line during passages.** Heatmap showing the MuAF level of 28 convergent SNVs for the indicated cell line during passages. Two types of mutations of P812R|L have been marked in red.


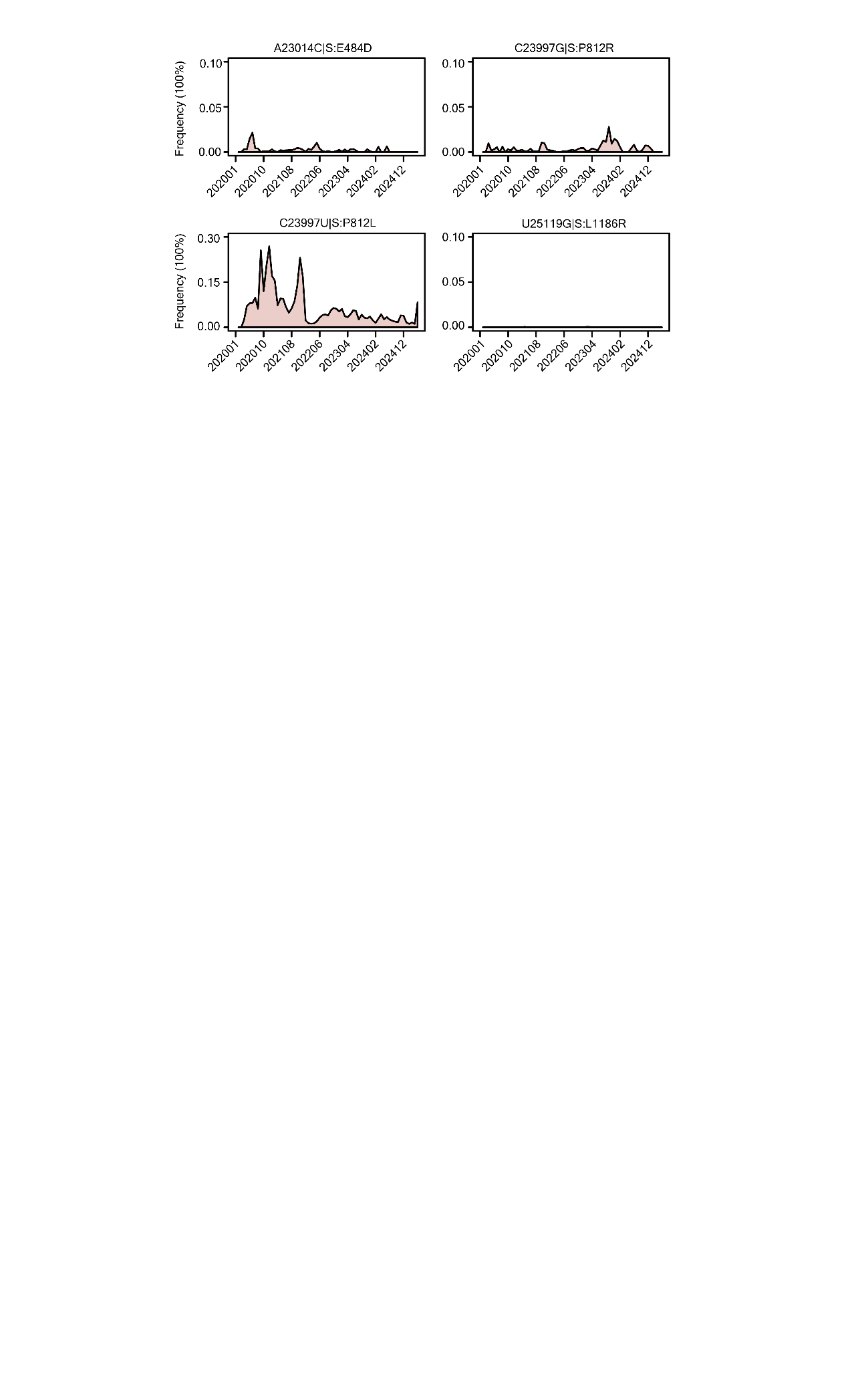


**Fig. S6. The population prevalence of the convergent SNVs found in six cell lines.** Line plots showing the populational prevalence over time for A23014C|S:E484D, C23997G|S:P812R, C23997U|S:P812L and U25119G|S:L1186R. The sequence data were obtained from the GISAID database (as of Jun 5, 2025).


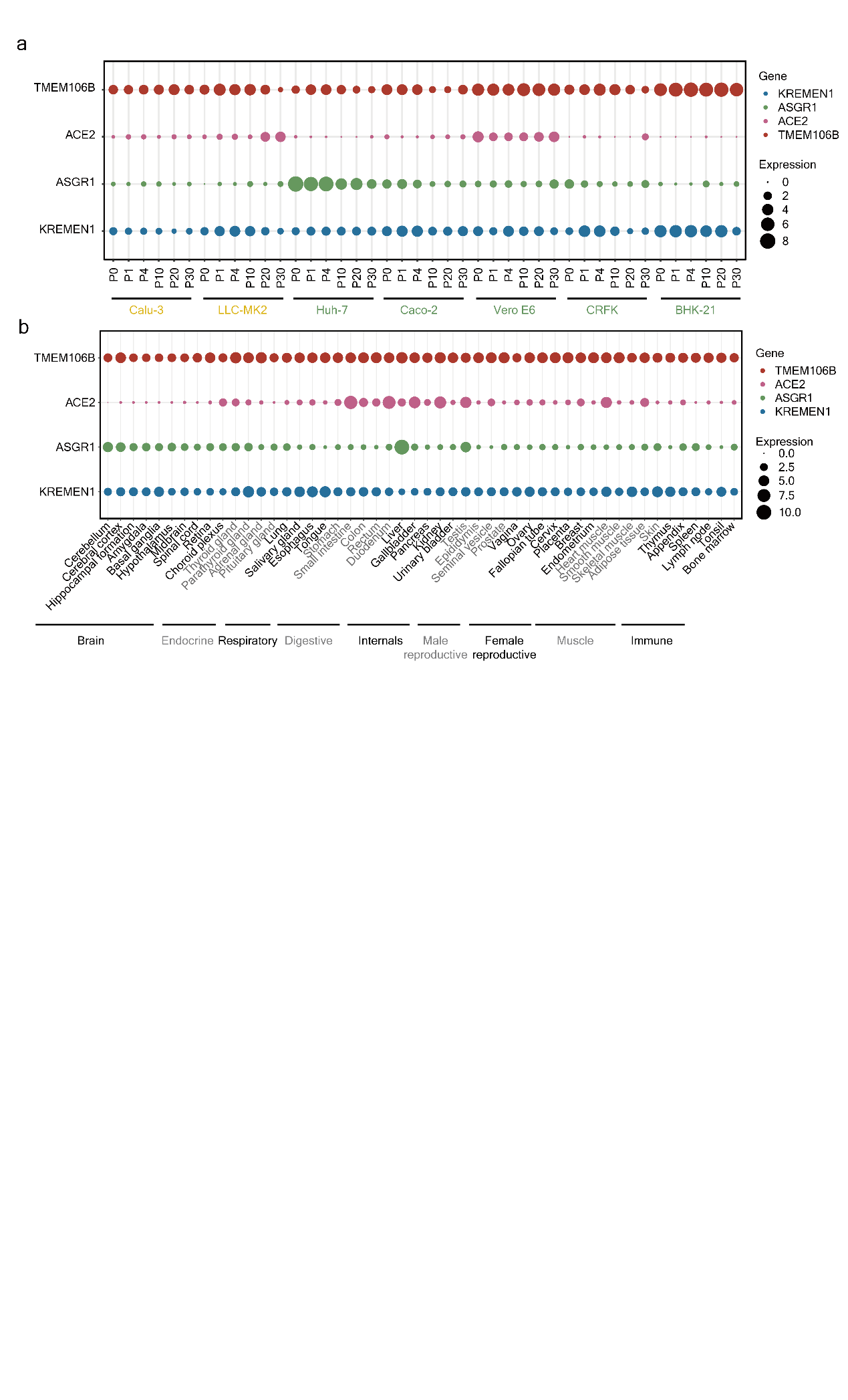


**
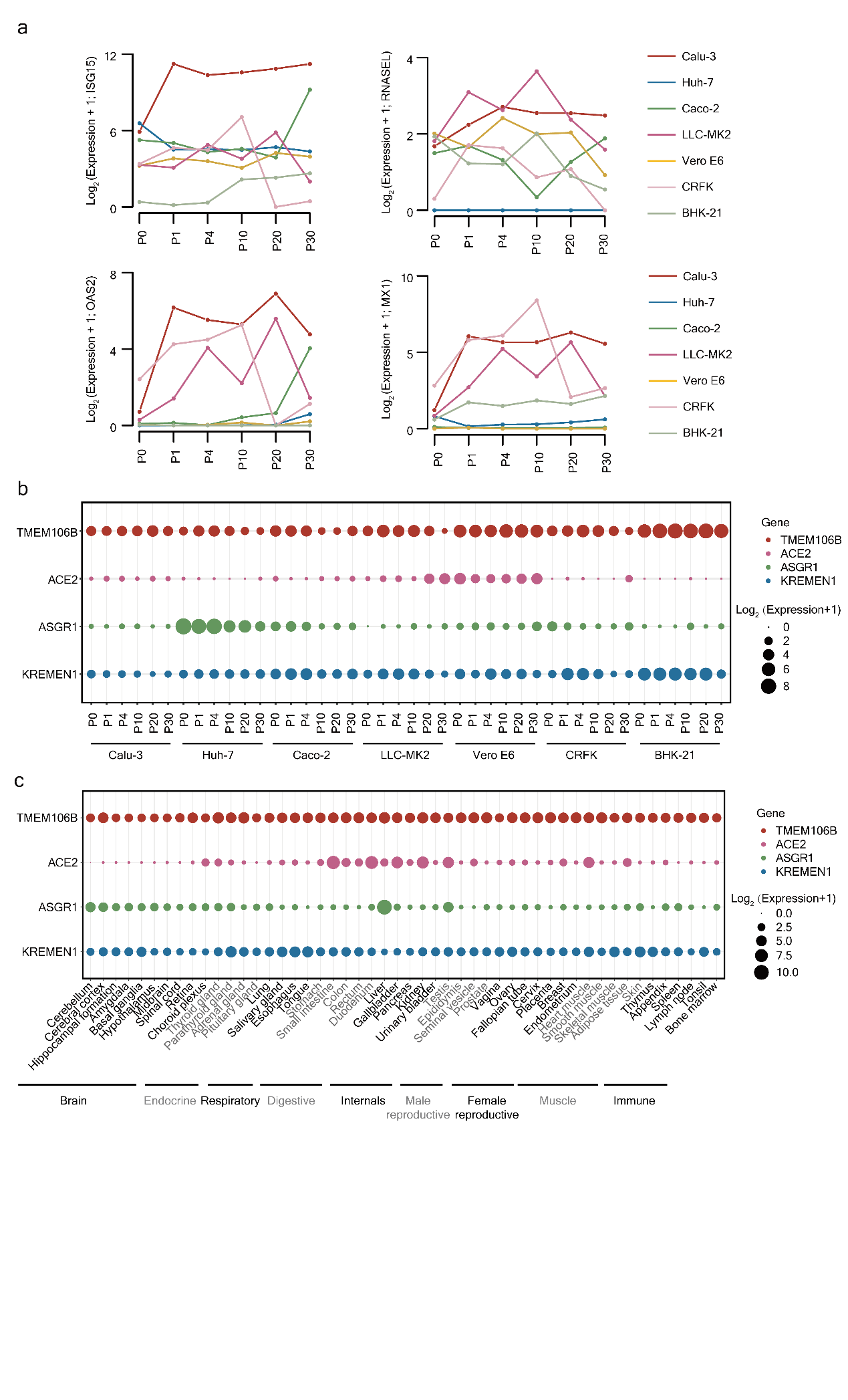
**

**Fig. S7.** **The expression level of representative ISGs and S-binding host factors. a**, Line plots displaying the RNA expression level of ISG biomarkers in the indicated cell lines. **b**, **c** Bubble plots showing the RNA expression level of S-binding host factors (ACE2, ASGR1, KREMEN1 and TMEM106B), in (a) the indicated cell lines and (b) the indicated tissues. RNA expression data of different cell lines was collected in this study, while data for different tissues was downloaded from the GTEx database. The size of each bubble represents the expression of genes quantified by RNA-seq.

**
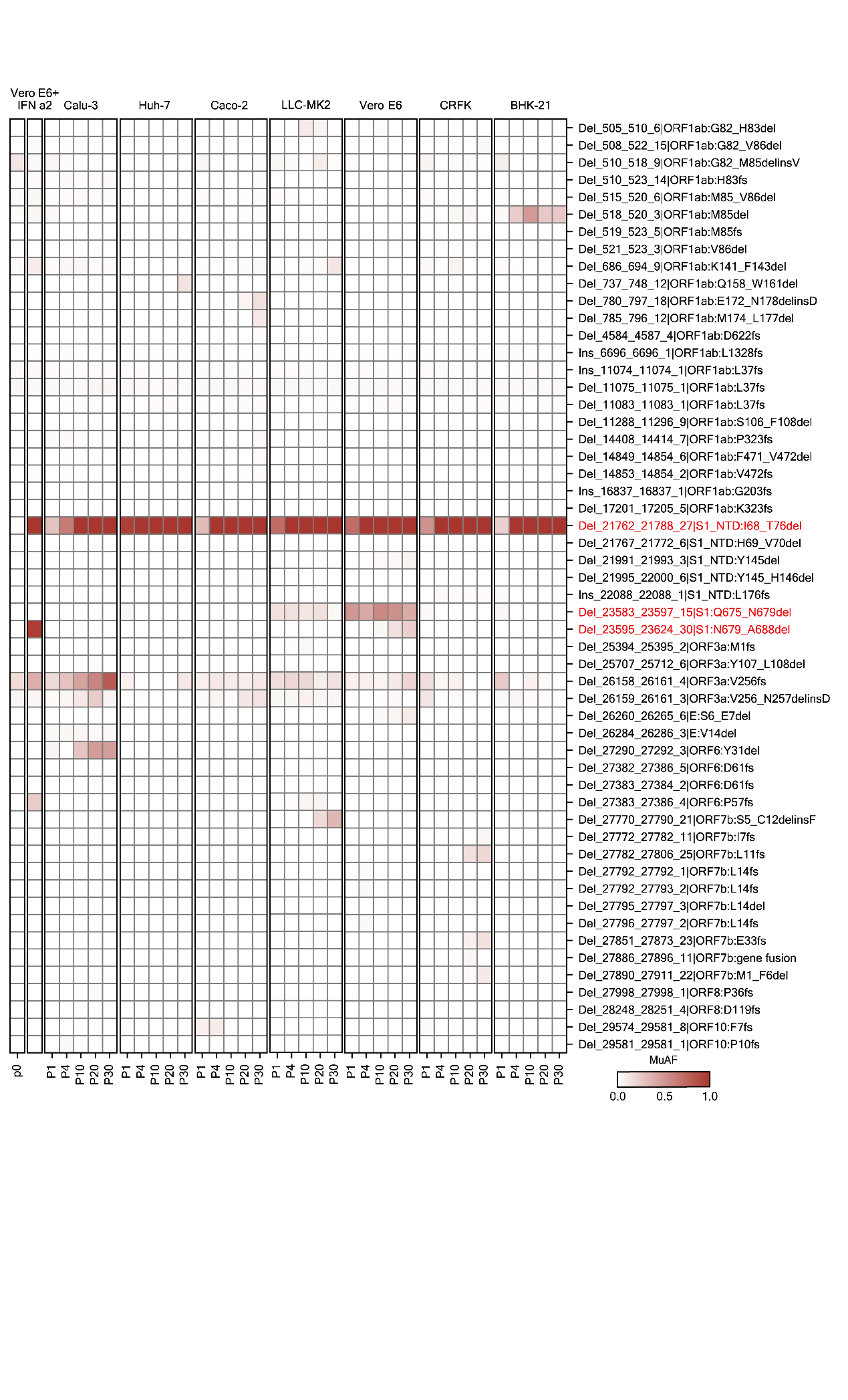
**

**Fig. S8. The MuAF level for the indels found in this study.** Heatmap showing the MuAF level of 54 indels for the indicated cell lines during passages. Q675_N679del, N679_A688del, and I68_T76del have been marked in red. The abbreviations Del, Ins, Delins and FS correspond to the following: deletion of nucleotide or amino acid, insertion of nucleotide or amino acid, frame-preserving deletion with amino acid loss and insertion, and frameshift mutation with the number of nucleotide inserted or deleted being not a multiple of 3.

| **Cell line** | **Organism** | **Tissue** | **Type or**  **morphology** | **SARS-CoV-2**  **susceptible** |
| --- | --- | --- | --- | --- |
| Calu-3 | Human (*Homo sapiens*) | Lung | Epithelial | Yes^1^ |
| Huh-7 | Human (*Homo sapiens*) | Liver | Epithelial | Yes^1^ |
| Caco-2 | Human (*Homo sapiens*) | Colon | Epithelial | Yes^1^ |
| Vero E6 | African green  Monkey (*Chlorocebus sabaeus*) | Kidney | Epithelial | Yes^2^ |
| LLC-MK2 | Rhesus macaque (*Macaca mulatta*) | Kidney | Epithelial | Yes^2,3^ |
| CRFK | House cat (*Felis catus*) | Kidney | Epithelial | Limited^3^ |
| BHK-21 | Hamster (*Mesocricetus auratus*) | Kidney | Fibroblast | Limited^1,2^ |

**Table S1. Overview of commercial cell lines used in study**

**Table S5. The initial SARS-CoV-2 used for different passaging studies**

| **Projects** | **SARS-CoV-2 strain** | | **SNPs for the initial strain** | **Lineages** | | **Cells (Passages for sequencing)** | |
| --- | --- | --- | --- | --- | --- | --- | --- |
| Chen et al.^4^ | WIV04 | / | | | Ancestral Virus | | Vero E6 (P0-P11); Huh-7 (P0-P9) |
| Ramirez et al.^5^ | DK-AHH1 | C241T,  C1059T\|ORF1ab:T265I,  C3037T\|ORF1ab:F924F,  G10465A\|ORF1ab:K3400K,  C14408T\|ORF1ab:P4715L,  C21742T\|S:S60S,  G22487A\|S:E309K,  A23403G\|S:D614G,  G25563T\|ORF3a:Q57H,  G28899T\|N:R209I | | | B.1 | | Vero E6 (P0, P2, P7, Day 42 postinoculation); Huh-7.5 (P0, P1, P5, P6) |
| This study | Strain T | C241T, C3037T\|ORF1ab:F924F, C14408T\|ORF1ab:P4715L, G18756T\|ORF1ab:P6164P, C18877T\|ORF1ab:L6205L, C22444T\|S:D294D, C22938T\|S:S459F, A23403G\|S:D614G, G25494T\|ORF3a:T34T, G25563T\|ORF3a:Q57H, C26735T\|M:Y71Y, C28854T\|N:S194L | | | B.1.36.1 | | Vero E6 (P0, P1, P4, P10, P20, P30); Huh-7 (P0, P1, P4, P10, P20, P30) |

**Table S6. The qPCR primer used in this study**

| **Primer name** | **Base sequence（5’-3’)** |
| --- | --- |
| SARS-CoV-2 N F | GGGGAACTTCTCCTGCTAGAAT |
| SARS-CoV-2 N R | CAGACATTTTGCTCTCAAGCTG |
| SARS-CoV-2 N Probe | AGCCGCCGCCTGGTCAACTCG |

**Reference**

1. Hoffmann, M. et al. SARS-CoV-2 cell entry depends on ACE2 and TMPRSS2 and is blocked by a clinically proven protease inhibitor. *Cell* **181**, 271-280 (2020).

2. Wurtz, N., Penant, G., Jardot, P., Duclos, N. & La Scola, B. Culture of SARS-CoV-2 in a panel of laboratory cell lines, permissivity, and differences in growth profile. *Eur. J. Clin. Microbiol. Infect. Dis.* **40**, 477-484 (2021).

3. Wang, L. et al. Susceptibility to SARS-CoV-2 of cell lines and substrates commonly used to diagnose and isolate influenza and other viruses. *Emerg. Infect. Dis.* **27**, 1380-1392 (2021).

4. Chen, Y., et al., Genetic mutation of SARS-CoV-2 during consecutive passages in permissive cells. *Virol Sin*. **36**, 1073-1076 (2021).

5. Ramirez, S., et al., Overcoming culture restriction for SARS-CoV-2 in human cells facilitates the screening of compounds inhibiting viral replication. *Antimicrob Agents Chemother*. **65**, p. e0009721 (2021).
